# Impact of Phytochemicals on PPAR Receptors: Implications for Disease Treatments

**DOI:** 10.1155/2022/4714914

**Published:** 2022-08-31

**Authors:** Ayesheh Enayati, Mobina Ghojoghnejad, Basil D. Roufogalis, Seyed Adel Maollem, Amirhossein Sahebkar

**Affiliations:** ^1^Ischemic Disorders Research Center, Golestan University of Medical Sciences, Gorgan, Iran; ^2^Discipline of Pharmacology, School of Medical Sciences, University of Sydney, Sydney, NSW, Australia; ^3^NICM Health Research Institute, Western Sydney University, Penrith, NSW, Australia; ^4^Department of Pharmacology and Toxicology, College of Pharmacy, Al-Zahraa University for Women, Karbala, Iraq; ^5^Department of Pharmacodynamics and Toxicology, School of Pharmacy, Mashhad University of Medical Sciences, Mashhad, Iran; ^6^Biotechnology Research Center, Pharmaceutical Technology Institute, Mashhad University of Medical Sciences, Mashhad, Iran; ^7^Applied Biomedical Research Center, Mashhad University of Medical Sciences, Mashhad, Iran; ^8^Department of Biotechnology, School of Pharmacy, Mashhad University of Medical Sciences, Mashhad, Iran

## Abstract

Peroxisome proliferator-activated receptors (PPARs) are members of the ligand-dependent nuclear receptor family. PPARs have attracted wide attention as pharmacologic mediators to manage multiple diseases and their underlying signaling targets. They mediate a broad range of specific biological activities and multiple organ toxicity, including cellular differentiation, metabolic syndrome, cancer, atherosclerosis, neurodegeneration, cardiovascular diseases, and inflammation related to their up/downstream signaling pathways. Consequently, several types of selective PPAR ligands, such as fibrates and thiazolidinediones (TZDs), have been approved as their pharmacological agonists. Despite these advances, the use of PPAR agonists is known to cause adverse effects in various systems. Conversely, some naturally occurring PPAR agonists, including polyunsaturated fatty acids and natural endogenous PPAR agonists curcumin and resveratrol, have been introduced as safe agonists as a result of their clinical evidence or preclinical experiments. This review focuses on research on plant-derived active ingredients (natural phytochemicals) as potential safe and promising PPAR agonists. Moreover, it provides a comprehensive review and critique of the role of phytochemicals in PPARs-related diseases and provides an understanding of phytochemical-mediated PPAR-dependent and -independent cascades. The findings of this research will help to define the functions of phytochemicals as potent PPAR pharmacological agonists in underlying disease mechanisms and their related complications.

## 1. Introduction

Peroxisome proliferator-activated receptors (PPARs) are a subfamily of the ligand-dependent nuclear receptor family. PPARs consist of three distinct subtypes, namely, peroxisome proliferator-activated receptor alpha (PPAR*α*), peroxisome proliferator-activated receptor gamma (PPAR*γ*), and peroxisome proliferator-activated receptor beta or delta (PPAR*β* or PPAR*δ*), each exerting specific biological activities depending on the particular targeting ligands and tissue localization [1–3]. They regulate a wide range of biological processes, including fatty acid metabolism, metabolic pathways, cellular differentiation, insulin sensitivity, cell migration, and inflammation. Therefore, PPARs can provide unique beneficial effects on cancer, atherosclerosis, metabolic diseases, cardiovascular diseases, neurodegeneration, reproduction, and inflammation via activation or inhibition of various up/downstream signaling pathways, including AMP-activated protein kinase (AMPK), mammalian target of rapamycin (mTOR), Sirtuins, and oxidative and inflammatory responses [1, 2, 4].

PPAR*α* is mainly known as a metabolic regulator which is expressed in liver and brown adipose tissue. It is associated with energy storage, lipogenesis, fatty acid up-regulation and *β*-oxidation, ketogenesis, gluconeogenesis, and inflammation in these tissues [1]. PPAR*β*/*δ* is involved in energy expenditure in fatty acid (FA) uptake, *β*-oxidation, placenta and gut development, inflammation reduction, cell proliferation, differentiation, cell survival, tissue repair, and energy homeostasis in muscle and white adipose tissue. It is ubiquitously observed in renal, gut, gastrointestinal tract, liver, and the central nervous systems [1, 2]. PPAR*γ* mediates energy storage-lipogenesis, glucose metabolism, and inflammation in white adipose tissue (WAT) and macrophages [1, 5]. Additionally, PPAR*γ* is an important target to treat several types of cancer, neurodegenerative diseases, long-chain fatty acid processing in the intestinal epithelium, body adiposity, mucosal defenses, and hypotensive and anticoagulant effects [1, 2, 4, 5]. Moreover, the PPAR*γ* isotype is expressed as two isoforms, PPAR*γ*1 and PPAR*γ*2. PPAR*γ*2 is expressed in adipose tissue, whereas PPAR*γ*1 occurs in adipose tissue, gut, vascular cells, brain, and special immune and inflammatory cells [1].

As a result of the broad and specific biological activities of PPARs, researchers have actively pursued the development of PPAR-targeting drugs. Some synthetic PPAR agonists, including thiazolidinediones (TZDs), pioglitazone, troglitazone, fibrates, glitazars, rosiglitazone, and gemfibrozil, were approved following several experimental and clinical studies [1, 2, 5]. Despite these advances, various studies have reported significant side effects of these PPAR agonists, such as heart failure, hepatotoxicity, fluid retention, edema, tumorigenesis, weight gain, and cardiotoxicity [2]. On the other hand, natural phytochemicals have shown promising potential as PPAR agonists, including endogenous unsaturated fatty acids, polyacetylenes, terpenoids, and polyphenols [3–6]. Hence, this review examines the impact of phytochemicals on PPAR receptors, with particular emphasis on the signaling pathways which PPARs enhance or inhibit in the management of various diseases. The mechanisms responsible for their toxicity are also discussed.

## 2. PPAR Mechanism of Action and Therapeutic Targets of Diseases

PPARs are involved in regulation of a wide spectrum of adverse reactions, including oxidative stress, inflammation, neuron degeneration, cardiovascular disease (CVD), multiple sclerosis (MS), Alzheimer's disease, diabetes, dyslipidemia, kidney dysfunction, gastrointestinal toxicity, cancer, autophagy, and immunity. This is associated with particular signaling pathways as well as the presence of specific coactivators/corepressors in each organ, such as inflammatory and antioxidant elements [1, 2, 6]. It is known that B-cell lymphoma 6 (BCL-6), the silencing mediator of retinoic acid and thyroid hormone receptor (SMRT), and the nuclear corepressor 1 (NCoR1) act as PPAR corepressors. Additionally, enzymatic coactivators modulate PPAR activity, including histone acetylase activity (steroid receptor coactivator 1 (SRC-1), cAMP response element-binding protein/p300), helicases (PPAR A–interacting complex (Pric)285), and an ATPase (SWItch/sucrose non-fermentable (SWI/SNF)). Additionally, nonenzymatic coactivators that bind to PPAR complex, such as PGC-1*α* (PPAR coactivator- (PGC-) 1*α*) and SMARCD1 (SWI/SNF related, matrix associated, actin-dependent regulator of chromatin subfamily d, member 1), have been reported [1, 6].

Mechanistically, PPARs heterodimerize with retinoid X receptors (RXRs) for binding to the peroxisome proliferator response elements (PPREs) as their upstream DNA binding site [1, 2, 6]. After a ligand binds to PPARs, and then making a heterodimer and binding to PPRE, PPARs regulate gene transcription by recruiting coactivators in transactivation, while they recruit corepressors in the transrepression of certain genes by activation of the heterodimer in the presence of RXR ligand. PPARs regulate gene transcription by recruiting coactivators in transactivation and coactivators/corepressors in the transrepression of certain genes. In transrepression function, PPARs recruit coactivators/corepressors and exert their negative regulation on certain genes by preserving or releasing corepressors, mitogen-activated protein kinase (MAPK) pathways, and physical interaction with transcription proteins (nuclear factor kappa B (NF-kB), Smad-3, activator protein 1 (AP-1), and signal transducer and activator of transcription (STAT)) and competing with target genes for binding their co-regulators [6]. Furthermore, PPARs show distinct functions in various pathways such as energy storage, modulating mTOR activity, flexible interaction with AMPK, regulation of insulin signaling and insulin sensitivity, tissue repair and remodeling, lipid metabolism, cell survival, and inflammatory cascades [1, 2, 6].

Although PPARs show mainly transcriptional activities (genomic action), they may also operate via the stimulation of nongenomic pathways (such as insulin-like growth factor- (IGF-) insulin receptor (IR), stress response, calcium influx, and MAPK). In light of these considerations, PPAR*γ* downregulates MAPK pathway as a main insulin/IGF axis cascade and reduces circulating insulin to prevent cell migration and proliferation [6]. In addition, PPAR*γ* can inhibit production of inflammatory cytokines by MAPK suppression in colon mucosal [1]. It can also decrease angiotensin II-induced proliferation in vascular smooth muscle cells (VSMCs) through diminishing c-fos and via blocking MAPK signaling pathways. Moreover, activation of PPAR*γ* suppresses MAPK pathway and its downstream signaling (Ets-1, matrix metalloproteinase (MMP)2, and MMP9) for inhibiting platelet-derived growth factor (PDGF) and thrombin-triggered VSMC migration [6].

**Figure 1:**
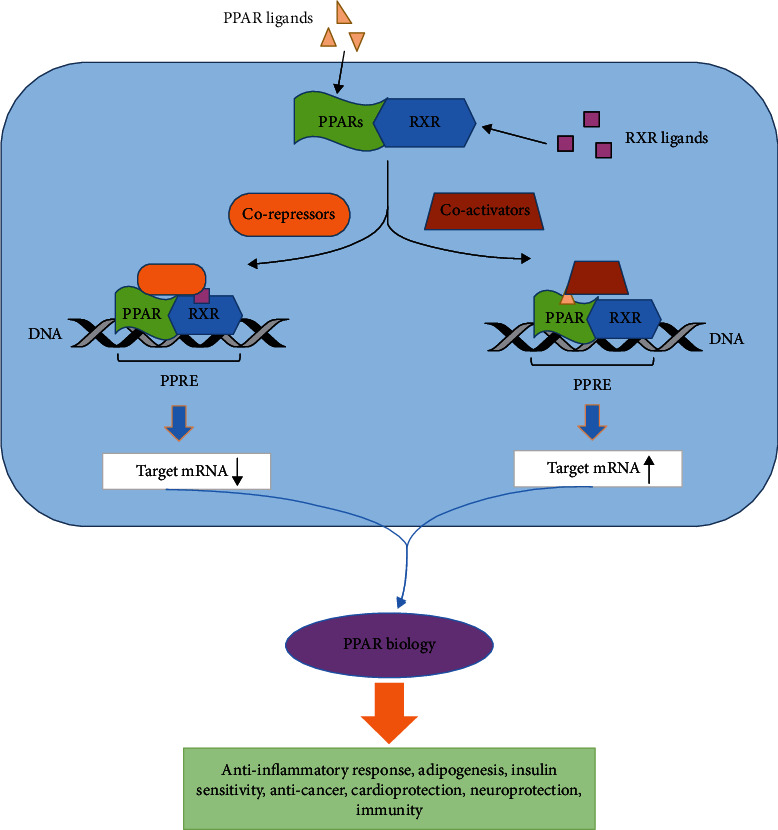
Concept map of PPARs icross-talk with RXR and PPRE.

It is therefore clear that PPARs play a critical role in management of diseases by genomic/nongenomic actions plus cross talk between PPARs and other key survival pathways and through their multiple functions with up-and downstream coactivators and co-regulators (Figure 1). To utilize these properties, multitask and safe PPAR agonists or antagonists are needed.

## 3. Methods

A systematic search strategy was developed to identify the impact of phytochemicals on PPAR receptors and the implications for the treatment of diseases. Searches were undertaken in PubMed, Scopus, and Google Scholar (January 2010 to March 2021). The terms “diseases,” “phytochemicals,” “herbal medicine,” and “PPARs receptor” were incorporated into an electronic search strategy. For each selected nutraceutical, the plausible mechanism of action was identified from the *in vitro* and *in vivo* evidence, and their clinically observed effects and relevant tolerability information were reported.

## 4. Phytochemicals with PPAR Modulation Activities

Plant-derived phytochemicals are well-known as modulators of the PPAR family, and their mechanisms in the prevention and treatment of human diseases have been ascribed to their physiological effects on carbohydrate and lipid metabolism. The versatile activities of phytochemicals are illustrated in Table 1 in terms of their PPAR activating abilities. The critical role of natural phytochemicals to human health in relation to their PPAR activating properties is discussed in the following section.

### 4.1. Curcumin

Curcumin is a natural lipophilic polyphenol from the rhizome of turmeric, *Curcuma longa* L. (Zingiberaceae), which can modulate a number of signaling pathways in its biological activities, including inflammation, atherosclerosis, and cardiovascular disease [7, 8]. Curcumin has been found to remarkably enhance peroxisome proliferator-activated receptor-*α* and *γ* (PPAR*α* and PPAR*γ*) in its anti-inflammatory, antioxidant, antihyperglycemic, and insulin sensitizer effects (Table 1) [8–10]. In this regard, it can initiate the PPAR*γ*/liver X receptor (LXR)/ATP-binding cassette transporter A1 (ABCA1) pathway by up-regulation of ABCA1, ATP-binding cassette transporters G1 (ABCG1), LXR*α*, scavenger receptor (class B) (CD36), and cytochrome P450 oxidase or sterol 27-hydroxylase (Cyp27); this then leads to reverse cholesterol transport and cellular cholesterol efflux in the prevention of hyperlipidaemia and atherosclerosis. In fact, curcumin can bind directly to PPAR*γ* or indirectly induce the production of intracellular ligands of PPAR*γ* [11]. Therefore, the induction of PPAR*γ* by curcumin could regulate glucose homeostasis and insulin resistance and also suppress inflammatory cytokines (including nuclear factor-*κ*B (NF-*κ*B) and matrix metalloproteinases (MMPs)) in macrophages and oxidative stress [9, 11]. Furthermore, curcumin drives PPAR*α* activation by regulating mitochondrial fatty acid *β*-oxidation, down-regulating sterol regulatory element-binding protein-1c (SREBP-1c) through suppression of LXR/RXR formation, inhibiting acyl-CoA:cholesterol acyltransferase (ACAT), interfering with NF-*κ*B and AP-1, and upregulating apolipoprotein A-I (Apo-AI), apolipoprotein A-II (Apo-AII), and mitochondrial 3-hydroxy-3-methylglutaryl-coenzyme A (HMG-CoA) reductase, thereby protecting against hypercholesterolemia and subsequent atherosclerosis [11, 12].

In addition, curcumin exerts an influence on metabolism through the activation of PPAR*γ* to ameliorate obesity/insulin resistance related disorders and certain inflammatory diseases. Some *in vitro* or *in vivo* studies indicated activity of curcumin on PPAR in the PPAR*γ* gene regulatory region is able to attenuate inflammation by inhibiting NF-*κ*B, tumor necrosis factor alpha (TNF-*α*), c-Jun N-terminal kinase (JNK), interferon gamma (IFN-*γ*), nitric oxide (NO), inducible nitric oxide synthase (*iNOS*), and AP-1. As well, antidiabetic properties of curcumin revealed through its antioxidant, anti-inflammatory, and antiapoptotic activities via mediation of PPAR*α*/*γ* lead to regulation of insulin signaling and phosphodiesterase/cyclic adenosine monophosphate (PDE/cAMP) in metabolism [13, 14]. Likewise, promoting PPAR*γ* ligand-binding activity by curcumin can stimulate free fatty acid catabolism, which can modulate glucose homeostasis, insulin resistance, and hemoglobin A1c (HbA1c) levels in related disorders such as diabetes and obesity [15]. Curcumin can also inhibit several inflammatory pathways and modulate obesity-related metabolic diseases by inhibiting low-density lipoprotein (LDL) and the level of intracellular cholesterol by activation of PPAR*γ*, leading to the suppression of *α*1 collagen, alpha smooth muscle actin (*α*-SMA), connective tissue growth factor (CTGF), transforming growth factor (TGF-*β*) receptors, platelet-derived growth factor subunit B (PDGF-*β*), interleukin-1 (IL-1), interleukin-13 (IL-13), and epidermal growth factor (EGF) [16]. Furthermore, molecular docking studies showed that curcumin as a PPAR*γ* agonist binds with Ile(341), Arg(288), Ser(289), Ala(292), Leu(333), Ile(326), Leu(330), and Met(329) amino acids in the active site of PPAR*γ* [8].

Curcumin has also demonstrated anticancer and apoptosis properties on many tumor cells. For instance, curcumin down-regulated the *β*-catenin/T-cell factors (Tcf) signaling pathway in the human colon cancer cell line HT-29, which leads to suppression of the expression of PPAR*δ*, 14-3-3*ε*, and vascular endothelial growth factor (VEGF) and subsequent induction of apoptosisin HT-29 cells [17]. In MCF-7 breast cancer cells, curcumin activated AMPK as an upstream signal of PPAR*γ* in 3T3-L1 adipocytes, resulting in the down-regulation of PPAR*γ* and a decrease in differentiation of adipocytes [18]. Furthermore, curcumin as a cancer therapy candidate is shown to exert its anticancer effect through PPAR*γ* activation and down-regulation of the aberrant WNT/*β*-catenin pathway leads to activation of glycogen synthase kinase-3*β* (GSK-3*β*), leading to the control of inflammation, proliferation, and angiogenesis in cancers [19]. Curcumin mediates its antifibrotic effects by the PPAR*γ* upregulation of matrix-degrading proteases, cathepsin B/L (CatB and CatL) [20]. Recently, it has been reported that curcumin mediates organic cation transporter 2 (OCTN2) expression through activation of the PPAR*γ*/RXR*α* pathway by binding to the peroxisome proliferator response elements (PPRE) in colorectal cancer SW480 cells [21].

Curcumin can suppress hepatic stellate cell (HSC) activation and modulate liver inflammatory injury by upregulation of PPAR*γ*, which can increase apoptosis or decrease cyclin D1 and proliferation to inhibit angiogenesis/cell growth, and also can cause a reduction in TGF-*β* signaling and extracellular matrix in regard to inhibition of HSC activation and liver fibrosis [22]. Much research shows that curcumin alleviates cholangiopathy and biliary fibrosis in multidrug resistance-2 gene (Mdr2^−/−^) mice via PPAR*γ* activation, TNF-*α* inhibition, and the stimulation of vascular cell adhesion molecule-1 (VCAM-1) expression in cholangiocytes [23]. Likewise, it can attenuate liver injuries by PPAR*γ* activation, the elevation of cellular glutathione (GSH) content, extracellular-signal regulated kinase (ERK) inhibition, and prevention of toll-like receptor 4 (TLR-4) expression leading to down-regulation of NF-kB in hepatic stellate cells [24]. Curcumin-low-molecular-weight PEGs (mPEG454) showed a therapeutic effect on dyslipidemia and nonalcoholic fatty liver disease via cAMP response element binding (CREB)/PPAR*γ*/CD36 pathway, by which the activation of CREB triggered inhibition of PPAR*γ* and CD36 expression in mediation of lipid homeostasis [25]. Meanwhile, curcumin improved lipid accumulation in nonalcoholic fatty liver disease via increasing PPAR*α* mRNA and protein levels in the liver and inhibition of DNA methylation at the PPAR*α* gene [26]. Thus, curcumin may prevent nonalcoholic steatohepatitis (NASH)/cirrhosis and nonalcoholic fatty liver disease through direct/indirect induction of PPAR*γ* expression [27].

In lung inflammation, curcumin acts as a mediator of inflammation and oxidative stress by the upregulation of PPAR*γ*, leading to the inhibition of TNF-*α* in acute lung injury and pulmonary diseases such as idiopathic pulmonary arterial hypertension [28]. PPAR*γ* activation by curcumin causes the upregulation of heme oxygenase-1 (HO-1) and blocks pulmonary cell proliferation, remodeling, differentiation, and apoptosis by mediating the protein kinase C (PKC)/AMPK/p38MAPK/NAD-dependent protein deacetylase (SIRT1)/PPAR*γ* pathway, and then, through attenuation of NF-*κ*B, signal transducer and activator of transcription-1 (STAT-1) and AP-1, protecting against lung inflammation [29].

In addition, curcumin can ameliorate renal fibrosis, a common pathology in chronic kidney disease, and arrest the cell cycle in the G1 phase. It seems that curcumin reduces fibroblast proliferation and extracellular matrix (ECM) accumulation through up-regulation of PPAR*γ* and down-regulation of Smad2/3-dependent TGF-*β*1 signaling [30]. Other studies indicated that curcumin inhibited TGF-*β*1-induced epithelial mesenchymal transition (EMT) via the ERK/PPAR*γ* signaling pathway in a Smad2/3-independent manner in renal tubular epithelial cells [25]. However, curcumin reveals its antifibrotic effect at the activation stage of renal fibrosis by reducing TGF/Smad, MAPK/ERK, and sphingosine kinase 1 (Sphk1)/sphingosine-1-phosphate (S1P), as well as increasing PPAR*γ* pathways to block fibrosis.

Growing evidence showed that curcumin exhibited a therapeutic effect in cardiometabolic syndrome treatment by an increase/activation of PPAR*γ* and suppressing the levels of inflammatory markers including NF-*κ*B, TNF-*α*, IL-6, and high-sensitivity C-reactive protein (hs-CRP) in both animal model and molecular docking [31]. Curcumin also inhibited myocardial cell necrosis and apoptosis by abrogating NF-*κ*B expression and stimulating expression of PPAR*γ* and B-cell lymphoma 2 (Bcl-2) in myocardial cells in a rat myocardial infarction model [32]. In vascular smooth muscle cells, curcumin diminished AngII-induced inflammatory factors and oxidative stress by enhancing PPAR-*γ* activity, leading to down-regulation of TNF-*α*, IL-6, NO, cell proliferation, p47phox, reduced nicotinamide adenine dinucleotide phosphate (NADPH) oxidase, and reactive oxygen species (ROS) production. These beneficial effects of curcumin enabled an explanation of its molecular mechanisms on atherosclerosis [33].

Previous studies have demonstrated the protective potential of curcumin on neurological diseases such as Alzheimer's disease, ischemic stroke, central nervous system (CNS) injury, chronic pain, trauma, multiple sclerosis, and Parkinson's disease. Curcumin alleviates neuroinflammation and the production of microglia, astrocytes, and inflammatory cytokines due to PPAR*γ* activation, leading to inhibition of amyloid-*β* accumulation as well as inflammatory signaling cascades such as Janus kinase (JAC)/signal transducer and activator of transcription (STAT), NF-*κ*B, and IL-12/IFN*γ* [34–37].

In addition, curcumin has immune-modulatory properties in various pathological or age-related diseases such as cancer, Alzheimer's disease, atherosclerosis, and metabolic disorders. It can enhance the immune system by the activation of PPAR*γ*, thereby decreasing the levels of proinflammatory cytokines (IL-1*α*, IL-1*β*, IL-12, IL-6, TNF-*α*, NF-*κ*B) and up-regulation of CD36, HO-1, and NADPH quinine oxidoreductase-1 (NQ-1) can occur, revealing an immunomodulatory effect of curcumin [35, 38, 39].

### 4.2. Resveratrol

Resveratrol, a natural polyphenol (stilbene) found in several plants such as grapes, peanuts, and other berries, has been reported to have antioxidant, anticancer, anti-inflammatory, cardioprotective, hypolipidemic, and metabolic regulation properties [40, 41], though therapeutic effects have been questioned in some clinical studies [41–43]. Previous studies indicated that resveratrol acts as a natural PPAR agonist onisotypes of PPARs and regulates metabolism [40, 41]. Resveratrol ameliorates atherosclerosis, platelet aggregation, lipid homeostasis, and total cholesterol accumulation through its antioxidant, anti-inflammatory, antiapoptotic, and lipid overload inhibition, and in addition improves endothelial function [42]. Interestingly, these effects have been shown to occur through activation of the PPAR*γ*/LXR*α* cascade, SIRT1, endothelial nitric oxide synthase (eNOS), AMPK, ABCA1, and G1, ERK1/2, inhibiting TNF*α*, IFN*γ* and NF-*κ*B, and promoting cholesterol efflux [40–42].

Resveratrol also ameliorates carboxymethyllysine- (CML-) induced pancreas damage and hyperglycemia through increasing insulin synthesis and upregulating pancreatic PPAR*γ* and pancreatic and duodenal homeobox-1 (PDX-1), as well as activating the nuclear factor erythroid 2-related factor 2 (Nrf2) pathway [43]. Resveratrol suppresses oxidative stress by activation of Nrf2 and PPAR*γ* signaling pathways and their crosstalk.

In diabetic cardiomyopathy, resveratrol inhibits myocardial fibrosis during hyperglycemic conditions by suppressing the ROS/ERK/TGF-*β*/periostin and TGF-*β*1/Smad3 pathways, along with modulating the SIRT1/CDK2-associated cullin 1 (CACUL1)/PPAR*γ* axis [42]. It seems that anti-inflammatory, antioxidant, antiapoptotic, and antifibrotic properties of resveratrol play a pivotal role in the up/down-regulations of the signaling cascades involved.

In further actions, resveratrol protects retinal pigment epithelium (RPE) cells from sodium iodate injury via its antioxidant and anti-inflammatory effects, leading to regulation of PPAR*α* and PPAR*δ* conformation and suppression of ROS and IL-8 production, as well as GSH up-regulation to attenuate oxidative stress and progression of age-related macular degeneration [44]. Furthermore, resveratrol in dyslipidemia or metabolic syndrome decreases body weight, regulates lipid deposition, modulates adipocyte gene expression, and stimulates white adipose browning, via phosphatidylinositol-3kinase (PI3K)/SIRT1, Nrf2, PPAR*γ*, TNF-*α*, and protein kinase A (PKA)/LKB1/AMPK signaling pathways [45]. Resveratrol exerts immunomodulatory effects through regulating PPAR*α*/RXR*α* activation, IL-10 signaling, natural killer cell signaling, leucocyte extravasation signaling, and IL-6 signaling, immune response pathways involved in disease [45]. Recently, a novel hybrid compound (PTER-ITC) was synthetized from trans-3,5-dimethoxy-49-hydroxystilbene (PTER), a natural dimethylated analog of resveratrol, and an isothiocyanate (ITC) conjugate. PTER-ITC revealed anticancer potential on breast cancer cell lines (MCF-7 and MDA-MB-231) through activation of PPAR*γ*, PPAR*β*,p38 MAPK, JNK, caspase 9, caspase 7, and caspase 3 pathways and downregulation of Bcl-2 and survivin [46]. Thus, resveratrol may be considered a natural PPAR agonist which qualifies as an effective candidate to prevent and treat a number of chronic diseases (Table 1).

### 4.3. Polydatin

Polydatin, also known as piceid, is a glycoside compound of resveratrol which exists in grape, *Polygonum cuspidatum*, *Fallopia japonica*, peanut, berries, and other sources [47–49]. Polydatin has shown biological activities, such as antagonist of platelet aggregation, cardioprotective, neuroprotective, hepatoprotective, antithrombotic, antiatherosclerotic, antitumor, antibacterial, protection of lungs, anti-inflammatory, antioxidant, nephroprotective, melanogenesis inhibitor, and immunostimulant [47, 48, 50–52]. Moreover, polydatin restored vascular endothelial cells (VECs) functions in high glucose conditions by PPAR*β*-NO signaling pathways which ameliorate diabetes-related cardiovascular diseases [52]. Polydatinin addition exerted antiatherosclerotic effects by Pre-B cell colony enhancing factor (PBEF) downregulation and activation of PPAR*γ* and SREBP-1, thereby regulating intracellular lipid metabolism in peritoneal macrophage, as well as decreasing cholesterol deposition and prevention of development of atherosclerosis [49, 50]. In diabetes mellitus- (DM-) associated liver disease, polydatin acts as PPAR*α*/*β* signaling pathway activator through its anti-inflammatory and antioxidant effects (Table 1) [53, 54]. To sum up, polydatin exerts a pronounced effect on oxidative stress and inflammatory-induced diseases through activation of PPAR subunits and associated signaling pathways.

### 4.4. Phlorotannins

Phlorotannins, polymers of phloroglucinol, are a group of polyphenolic bioactive compounds which were found in brown alga [55, 56]. They possess several biological activities including antimicrobial, antiviral, hepatoprotective, cardioprotective, anti-inflammatory, neuroprotective, anticarcinogenic, immunomodulatory, hypolipidemic, antidiabetic, and antioxidant properties [55–57]. *Ecklonia*, a genus of kelp and brown alga which has abundance of phlorotannins, especially of the eckol-type, has hepatoprotective activity by increasing PPAR*α* and carnitine palmitoyl-transferase 1 (CPT-1) along with decreasing SREBP-1 and triglyceride (TG) to prevent fatty acid oxidation and reducing lipogenesis in ethanol-induced fatty liver [57]. Furthermore, phloroglucinol compounds of the aerial parts of *Potentilla longifolia* Wild. Ex Schlecht. protected 3T3-L1 adipocyte cells against lipid accumulation by downregulating SREBP1c, fatty acid synthase (FAS), stearoyl CoA desaturase-1 (SCD1), glycerol-3-phosphate acyltransferase (GPAT), PPAR*γ*, and CCAAT-enhancer-binding protein *α* (C/EBP*α*) adipogenesis-related proteins [58]. Although phlorotannins demonstrated several beneficial effects, there was evidence of side effects or toxicity in cell lines, both in animal and human studies. However, further studies should evaluate safety and toxicity of phlorotannins for use as functional foods and pharmaceuticals (Table 1) [59].

### 4.5. Quercetin

Quercetin is a common and important flavonoids that is widely distributed in tea, onions, peppers, plums, mangos, and various types of berries, fruits, and vegetables [60–62]. Quercetin plays an important role in anti-inflammation, antioxidation, antiviral, anticancer, antiatherosclerotic, cardioprotection, and other biological activities in the prevention and treatment of diseases [60–62]. Quercetin can inhibit atherosclerosis-induced myocardial infarction (MI), heart failure, and hypertension by upregulation of PPAR*γ* and the signaling cascades involved, including the antioxidant pathway and the downregulation of inflammatory cytokines (Table 1) [60–66]. However, the PPAR*γ*2 chemically activated luciferase gene expression (CALUX) culture study showed that quercetin (10 *μ*M) co-incubated with vitamin C (500*μ*M, to prevent auto-oxidation) can potentially increase the effect of PPAR*γ* ligands and expression of PPAR*γ*-cellular receptors leads to synergistic effects with endogenous PPAR*γ* agonists [67].

In metabolic disorders such as obesity and metabolic syndrome, quercetin can enhance WAT browning and brown adipose tissue (BAT) activation due to activation of *β*3-adrenergic receptor (*β3AR)*/PKA/AMPK/PPAR*γ*/peroxisome proliferator-activated receptor gamma coactivator 1-alpha (PGC1*α*) pathways, by this means inducing expression of uncoupling protein 1 (UCP1) and ABCA1 to promote adenosine triphosphate (ATP) and inhibit fat accumulation [68–70]. Owing to its relevance in adipogenesis, it appears that the inhibition of PPAR*γ*, C/EBP*α*, or SREBP plays a pivotal role in obesity treatment. Furthermore, quercetin may exert its antidiabetic and glucose uptake effects through activating SIRT1/ PPAR*γ*/AMPK signal cascades to improve the complications of insulin resistance and diabetes [71]. The combination of quercetin (0.1 *μ*M) and pioglitazone (0.1 *μ*M, a PPAR*γ* agonist) inhibited the angiotensin II (Ang II)-induced contractile effect in fructose-streptozotocin (FSTZ)-diabetic rats via antioxidant and NO release properties [72]. In another study, quercetin showed anti-diabetic effects more than antiobesity effects in high-fat high-sucrose diet (HFHSD) animals which consumed quercetin (30 mg/kg/BW/day) for 6 weeks. Likewise, lipogenic enzymes and lipoprotein lipases, including acyl-coenzyme A oxidase (ACO), CD36, carnitine palmitoyltransferase-1b (CPT-1b), PPAR*α*, PGC-1*α*, uncoupling protein 3 (UCP3), transcription factor A mitochondrial (TFAM) and cyclooxygenase-2 (COX-2), remained unchanged in adipose tissue, while quercetin treatment reduced fructosamine, basal glucose, insulin and homeostatic model assessment for insulin resistance (HOMA-IR), as accepted diabetic markers in rat models [73].

PPAR isoforms have gained significant attention in CVD treatment. Quercetin exhibited antiatherosclerosis effect by upregulating PPAR*γ*/LXR*α*/ ABCA1 and promoting cholesterol efflux in THP-1 derived foam cells [62]. Moreover, the administration of quercetin reduces ischemia/reperfusion injury by upregulating SIRT1//PPAR*γ*/PGC-1*α*, activating PI3K/Akt pathway, suppressing myonecrosis, increasing Bcl-2/Bax (pro-apoptotic protein), inhibiting the inflammatory cascade, scavenging ROS, and enhancing cardiac function [61, 66]. Therefore, quercetin, by increasing or activating PPAR*γ* and associated signaling cascades in the heart, exerts cardioprotective effects in CVDs, including hypertension, heart failure, ischemia, and atherosclerosis due to antioxidant, anti-inflammatory, and antiapoptotic disease [60–66]. Also, quercetin inhibited activation of all three isoforms of PPAR through its anti-inflammatory and antioxidant properties in obesity-related disorders and inflammatory diseases and an enhanced immune system [74]. Likewise, quercetin displayed its beneficial effects such as lipid lowering and suppression of the lipid accumulation-induced chronic inflammation by the PPAR*α* cascade in cultured chicken hepatocytes [75]. Furthermore, quercetin treated neurodegenerative dysfunction in the mouse Parkinson's disease model through up-regulating PPAR*γ*, PGC-1*α*, and TFAM to activate the polycystin 1 (PKD1)/Akt pathway [75].

### 4.6. Kaempferol

Kaempferol is a flavonol that is abundant in fruits, vegetables, and various medical plants, such as grapefruit, tea, and berries [76, 77]. Numerous studies have supported diverse beneficial properties of kaempferol, including antioxidant, anti-inflammatory, anticarcinogenic, antiobesity, antiatherosclerotic, cardioprotective, antihyperlipidemia, antiosteoporotic, and antidiabetic and estrogenic/antiestrogenic activities [76–79]. In addition, it reduced cholesterol, glucose, and TG levels through liver X receptor (LXR) activation and inhibition of sterol regulatory element-binding proteins (SREBPs), and without the side effect of hepatic steatosis [76–80]. Kaempferol also enhanced the expression of ACO, cytochrome P450 - family4 – subfamily a - polypeptide 1 (CYP4A1) and PPAR*α*, thereby reducing fat and lipid accumulation in obesity [79]. Published data revealed that in metabolic disorders, especially obesity and fat, kaempferol increased PPAR*α*, PPAR*δ*, and target genes, thereby inducing autophagy and fatty acid uptake as well as decreasing PPAR*γ* and SREBP-1c expression via activation/inhibition of related signaling pathways regulating obesity and metabolic dysfunctions (Table 1) [76–82]. Although beneficial antioxidant and anti-inflammatory effects of kaempferol have been reported, the precise molecular target and mechanism of kaempferol in the treatment of diseases remains unclear. Therefore, further study is needed to investigate the kaempferol mechanisms of action.

### 4.7. Rutin

Rutin, quercetin-3-O-rutinoside, is a flavonol with significant beneficial properties, such as antioxidant capacity, anticarcinogenic, cardioprotective, antiatherosclerotic, antiadipogenic, neuroprotective, and antihyperuricemia activities [83–89]. A number of *in vitro* and *in vivo* studies indicated that rutin can improve glucose uptake, hyperlipidemia, insulin resistance, lipid accumulation, obesity, and metabolic dysfunction through modifying the expression of PPAR*γ* and SREBP-1cin adipose tissue, thereby promoting AMPK and Akt activities to regulate body fat deposition [83–87]. Also, rutin attenuated NOD-like receptor family pyrin domain containing 3 (NLRP3) inflammasome activation through its anti-inflammatory and antioxidant effects in response to fructose-induced renal hyperlipidemia and injury [88]. Likewise, rutin, by stimulating insulin (Akt and ERK1/2) pathways and inhibiting leptin (JAK2/STATE3) cascades, triggered PPAR*α*, carnitine palmitoyl-transferase 1 (CPT1), and organic cation transporter 2 (OCTN2) up-regulation, resulting in renal urate and lipid lowering [88]. Moreover, rutin exhibited neuroprotective effects due to its ability to retard oxidative stress in brain tissue by stimulating PPAR*δ* (an abundant PPAR isoform in neural tissue and brain), leading to a promotion of antioxidant systems, including glutathione peroxidase (GPX), GSH, and paraoxonase (PON-1, PON-3) and a reduction of PON-2 in the cisplatin-neurotoxic rat model [89]. Taken together, rutin attenuated the metabolic dysfunction or other diseases induced by oxidative/inflammation stress through stimulation or inhibition of molecular mechanisms associated with a regulation of PPAR*α*/PPAR*γ*/PPAR*δ* levels (Table 1).

### 4.8. Hesperetin

Hesperidin and its aglycone hesperetin, a methoxylated flavanone known as citrus flavonoid, have particular pharmacological activities associated with high permeability in cell membranes, such as anti-inflammatory, antioxidant, antihypertensive, cardioprotective, vasodilation, anticancer, immunomodulator, antiallergic, neuroprotective, antiepileptic, antidepressant, lipid lowering, capillary fragility-reducing, antiadipogenic, and PPAR*γ* agonist properties (Table 1) [81, 90–98]. Furthermore, hesperidin/hesperetin exerted their beneficial effects through PPAR*γ* activation and subsequently modulating both PPAR*γ*-dependent/independent pathways in targeted tissue [90, 98].

These studies indicated that hesperidin restored oxidative stress and inflammation-induced hepatotoxicity via boosting hepatic PPAR*γ* expression and antioxidant markers, as well as reducing liver function enzymes and inflammation cytokines [92, 97]. Also, hesperidin/hesperetin stimulated PPAR*γ*, which is centrally involved in the mediation of antiapoptotic (diminishing JNK, caspase-3/9, p53, Bax), anti-inflammatory (attenuating TNF-*α*, IL-1*β*, IL-6, monocyte chemoattractant protein-1 (MCP-1), intracellular adhesion molecule-1 (ICAM-1)), and antioxidant (increasing superoxide anion dismutase (SOD), catalase (CAT), GSH) effects and improving inotropic and lusitropic cardiac function (rate of left ventricular systolic pressure (+dP/dt), rates of pressure fall (-dP/dt), mean arterial pressure (MAP)) in rat heart hypertrophy and IR models [93–95].

Interestingly, hesperidin showed antiadipogenic and delipidating effects by inhibiting *PPARγ*, CCAAT-enhancer-binding protein *β* (*C/EBPβ*), *SREBP1-C*, and *perilipin*, that are involved in different stages of adipogenesis (lipolysis and lipogenesis). In addition, it increased adipose triglyceride lipase in preadipocytes derived from human mesenchymal stem cells but also acted as a PPAR*γ* agonist and increased C/EBP*α* to decrease insulin and lipid in the 3T3-L1 adipocytes model [81, 90, 91]. It can be postulated that hesperidin/hesperetin, as a PPAR*γ* agonist, leads to attenuation of the inflammatory response and is thus ultimately protective against diseases through activation of radical scavenging activity.

### 4.9. Apigenin

Apigenin, a flavone abundant in foods injested daily, such as fruits, vegetables, and some medicines, possesses various biological activities including antioxidant, anti-inflammatory, anticancer, antihyperglycemic, antiadipogenic, antiobesity, cardioprotective, antifibrotic, antidepressant, antidiabetic, and hepatoprotective actions [99–103]. Moreover, apigenin can also downregulate PPAR*γ* and CEBP-*α* in the early phase of adipogenesis in 3T3-L1 adipocytes and protect against high-fat diet- (HFD-) induced metabolic syndrome in rats. Apigenin also prevents lipid accumulation and enhances adipocyte differentiation, thereby having hepatoprotective effects [99–103]. Recent research has established that apigenin is a PPAR modulator that inhibits obesity-induced metabolic syndrome via suppressing PPAR*γ* and PPAR*α*, resulting in activation/inhibition of upstream or downstream targets, such as STAT3, C/EBP-*α*, SREBP-1c, CD36, and Nrf2 in adipose tissues [99, 100, 103]. In addition, another study showed that apigenin provoked expression of PPAR*γ* in the macrophage to reduce metabolic abnormality and liver/muscular steatosis in HFD and diabetic rat [101]. Likewise, apigenin attenuated carbon tetrachloride (CCl_4_)– and bile duct ligature (BDL)–induced liver fibrosis by alleviating autophagy and activated hepatic stellate cells (HSCs) and extracellular matrix (ECM) formation via activating PPAR*α* and inhibiting TGF-*β*1/Smad3 and p38 pathways [102]. However, to further confirm the precise underlying mechanisms of apigenin on PPARs specifically in adipose, macrophage, or other tissues, *in vivo* models of obesity and ob/ob *in vitro* studies are needed.

In the cardioprotective effects of apigenin, previous studies reported that PPAR*α* and PPAR*γ* were involved in ameliorating cardiac hypertrophy and myocardial abnormality [104, 105]. Herein, apigenin in diabetic rats increased PPAR*γ* to attenuate MI-induced myonecrosis and cardiac dysfunction [105]. In renovascular hypertensive rats, it improved cardiac hypertrophy and glucolipid metabolism by directly inhibiting angiotensin II and hypoxia inducible factor-l*α* (HIF-1*α*), and subsequently diminishing PPAR*γ* and increasing PPAR*α* led to modulation of myocardial CPT-1, pyruvate dehydrogenase lipoamide kinase isozyme 4 (PDK-4), glycerol-3-phosphate acyltransferase (GPAT),and glucose transporter-4 (GLUT-4) proteins [104]. Furthermore, apigenin by its antioxidant and anti-inflammatory properties activated PPAR*γ* to protect against depression or mice pulmonary fibrosis by decreasing NLRP3 inflammasome, microglia, malondialdehyde (MDA), and apoptosis [106] or TGF-*β*1, matrix metallopeptidase 9 (MMP-9), and vimentin [107] in rat depression or mouse pulmonary fibrosis models, respectively. Therefore, the pharmacological effect of apigenin on PPARs suggests a novel approach in the treatment of cardiovascular, brain/nervous system, and immunity complications (Table 1).

### 4.10. Naringenin

Naringin (a flavanone glucoside) and naringenin (its aglycone) are major flavonoids of citrus fruit, grapefruit, tomato, and orange with various pharmacological activities, such as antioxidant, anti-inflammatory and antihypercholesterolemia, antiobesity, hypotensive, cardioprotective, neuroprotective, and metabolic syndrome therapy [108–113]. Naringenin improved metabolic disturbances via PPAR*α* and/or PPAR*γ* up-regulation and stimulation (PGC1*α*, CPT-1, UCP1, UCP2)/suppression (LXR*α*, adipogenic, lipogenic) of its related underling up/downstream kinases, enzymes, genes, and receptors, thereby providing antioxidant and anti-inflammatory effects in diabetic, hypercholesterolemia, obesity, and lipid metabolism liver dysfunction models, as shown in Table 1 [109–111, 114–117]. Some researchers have reported naringenin as a PPAR*α*/*γ* agonist [108, 111], but using naringenin supplementation had no significant effect on PPAR*α*/*γ* (slightly decreased) in ovariectomy-induced metabolically disturbed female mice. Interestingly, it increased fatty acid oxidation (CPT1*α*) and lipogenesis *de novo* (SREBF1) but decreased acyl-CoA oxidase 1 (ACOX1), another fatty acid oxidation target [108]. Also, in a further study, naringenin blocked expression of adipogenic and lipogenic activity by inhibiting LXR*α*/SREBP1c/PPAR*γ* signaling cascade to restore hepatic lipid accumulation and liver dysfunction in HBx-induced hepatic steatosis [118].

PPAR isoforms (*α*, *β*, and *γ*) seem to have pivotal actions in cardiac and renal injuries. Naringenin, through the activation of PPAR*α*, PPAR*β*, and PPAR*γ*, ameliorated diabetic nephropathy and cardiomyocyte hypertrophy, which was associated with an increase in CYP4A-20-Hydroxyeicosatetraenoic acid (20-HETE), cytochrome P450-family2-subfamily j-polypeptide 3 (CYP2J3), and 14,15-epoxyeicosa-5,8,11-trienoic acid (14,15-EET) levels, respectively [112–119]. Thus, naringin/naringenin may be effective as a potential complementary/alternative medicine PPAR modulator in the treatment of immune, brain, cardiac, metabolic, and renal diseases.

### 4.11. Catechins

Catechins are a large group of flavonoids, with flavan-3-ol structure, including catechin, epi-catechin, epigallocatechin, epigallocatechin-3-gallate, and proanthocyanidins found in many plants and also dietary foods such as apples, tea, cocoa beans, grape seed, and red wines [120–130]. Notably, catechins have multibeneficial biological effects, for instance antiobesity, lipid lowering, antioxidant, anti-inflammatory, antidiabetic, anticancer, antiatherosclerotic, cardioprotective, neuroprotective, and nephroprotective [120–130]. (-)-Epigallocatechin-3-gallate (EGCG), a green tea catechin, exhibited PPAR*α* and PPAR*γ* agonist properties in subcutaneous adipose tissues, but PPAR*γ* antagonist activity in epididymal adipose tissue to reduce obesity and epididymal white adipose tissue weight in HFD mice via activation of AMPK [120]. In addition, EGCG and catechins suppressed differentiation of adipocyte by reducing ROS, inflammation, insulin signaling, and the stress-dependent mitogen-activated protein kinase (MAPK) kinase, (MEK)/ERK, and PI3K/Akt pathways. Additionally, increasing cyclic adenosine monophosphate (cAMP)/PKA signaling led to inactivation of PPAR*γ*, C/EBP*α*, and forkhead transcription factor O1 (FoxO1) as clonal expansion-related genes in 3T3-L1 cells or preadipocyte models [121, 123–126]. Interestingly, procyanidin B2 (a catechin type) activated PPAR*γ* to regulate macrophage M2 polarization and manipulation of M1/M2 macrophage homeostasis in metabolic inflammatory diseases. Likewise, it induced M2 macrophage markers, including arginase (Arg1), Ym1, found in inflammatory zone (Fizz1) and cluster of differentiation 206 (CD206^+^) as well as PPAR*γ* targets (CD36, ABCG1), but inhibited the M1 markers in diabetic mice macrophages [122].

EGCG exerted its beneficial anticancer effects via PPAR*α* activation and inactivation of HO-1/Nrf2 pathway on some cancer cell lines, including pancreatic, esophageal, MCF-7, and ovarian. However, as a consequence of EGCG-induced PPAR*α* expression, HO-1 is negatively regulated by PPAR*α* as its direct target, depending on cell type and ligand stimulation. Therefore, PPAR*α* activation attenuates EGCG-induced HO-1 up-regulation and sensitizes cancer cells to EGCG [127]. In addition, catechins activated PPAR*γ* via their anti-inflammatory, antioxidant, and antiapoptotic effects to ameliorate cardiac, renal, brain, and nervous system injuries induced by their related diseases [128–130]. Additionally, catechins appeared to be critical regulators of PPARs (PPAR*α*, PPAR*γ*, PPAR*α*/*γ*, and PPAR*δ*) (Table 1) that are involved in protection of organs, and by inhibiting/stimulating their upstream or downstream targets improved each of the organ functions [129–131].

### 4.12. Berberine

Berberine is an isoquinoline alkaloid, which exists in plants such as *Berberis* spp. and *Rhizoma coptidis*. In addition, several previous studies have reported that berberine is considered anti-inflammatory, antidiabetic, cardioprotective, neuroprotective, antihyperlipidemic, antioxidant, hepatoprotective, and antiadipogenic potential [132–138]. Berberine exhibited its pharmacological effects through PPARs, especially as selective PPAR*α* agonist in regulation of metabolic, liver, renal, cardiac, and brain dysfunctions (Table 1) [135, 137, 139–142]. Berberine affects upstream or downstream signaling targets, resulting in activation of PPAR*α*, thereby reducing lipogenesis and promoting *β*-oxidation in animal metabolic dysfunction models [133–135, 137]. Interestingly, berberine activated PPAR*α*/nitrous oxide systems (NOS)/NO signaling pathway in cardiac animal experiments, which indicated that NO is a pivotal downstream target of PPAR*α* signaling cascade in cardiachypertrophy [140, 141].

### 4.13. Cinnamic Acid

Cinnamic acid is an organic and aromatic unsaturated plant-based carboxylic acid (with two cis and trans isoforms) exerting beneficial therapeutic effects such as antitumoral activity, antioxidant, anti-inflammation, antiatherogenic, hepatoprotection, cardioprotection, and neuroprotection [143–145]. Cinnamic acid exhibited a PPAR*α* agonist role to reduce lipid accumulation and neurodegeneration in cellular and animal models [143–145]. Interestingly, it acted as PPAR*γ* antagonist, resulting in inhibition of hepatic lipogenesis and fatty acid intake in HepG2 cells and *db/db* mice (Table 1) [144]. A recent study indicated that poly lactic-co-glycolic acid (PLGA) nanoparticle of cinnamic acid at concentration of ≥25 *μ*M inhibited MCF-7 cellular proliferation via PPAR*γ* signaling pathway, leading to a drop of metabolic activity and Ki-67 antigen to exert its cytotoxic effects on breast cancer [146]. Thus, cinnamic acid can act as agonist or antagonist of PPARs to regulate abnormality of various diseases.

### 4.14. Glycyrrhizic Acid

Glycyrrhizic acid (Glycyrrhizin) is a bioactive triterpenoid that was extracted from *Glycyrrhizaglabra* L. roots [147, 148]. Previous studies reported beneficial effects of glycyrrhizinin treatment of diseases and some research investigated the relationship of glycyrrhizic acid and PPARs (Table 1) [147–149]. In addition, new synthetic derivatives of glycyrrhizic acid, 2-cyano-substituted analogues, and 19 glycyrrhetic acid exhibited promising potential for PPAR*γ* activation to inhibit HT-29, HCT-15, MCF-7, and HepG2 carcinogen cell lines [150]. In addition, 19 glycyrrhetic acid derivative increased PPAR*γ* and reduced MMP-2/MMP-9 to act as antitumor agent against MCF-7 cells [150]. In another study, intraperitoneal injection of 50mg/kg glycyrrhetic acid in male Sprague-Dawley rats fed *ad libitum* with standard diet improved insulin sensitivity, reduced lipid (total cholesterol (TC), LDL, and triacylglycerol (TAG)), up-regulated PPAR*α* and PPAR*γ* in the liver, and revealed antiglucocorticoid effects [151]. Finally, glycyrrhetic acid exerts a role as PPAR*α*/*γ* agonist due to its antioxidant and anti-inflammatory properties.

### 4.15. Oleanolic Acid

Oleanolic acid is a natural pentacyclic triterpenoid found in medicinal plants, fruits, and vegetables [152, 153]. It showed some pharmacological potential through its dual agonist actions on PPAR in tissues [153, 154]. Likewise, oleanolic acid simultaneously activated PPAR*γ*/*α*, leading to an increase of fatty acid transport protein 1 (FATP-1) and long-chain acyl-CoA synthetase (ACSL) to regulate metabolic dysfunction in 3T3-L1 and C2C12 cells [154]. Also, oleanolic acid operated as a ligand of PPAR*γ*-1 or PPAR*δ* for management of obesity or high glucose-induced metabolic abnormality in animal and cell line models [152, 153]. However, in the *in vivo* studies, it had cardioprotective and hepatoprotective effects by stimulation of PPAR*α* and PPAR*γ*, respectively [155, 156]. In another study, isolated oleanane-type triterpenoid of *Pulsatilla koreana* root showed anti-inflammatory effects via activation of PPAR binding to PPRE luciferase reporter, thereby inducing an inhibition of NF-*κ*B, iNOS, and ICAM-1in HepG2 cells (Table 1) [157]. However, future studies are needed to identify the precise mechanism of the PPARs agonist role of oleanolic acid.

### 4.16. Ursolic Acid

Ursolic acid (UA), a pentacyclic triterpenoid that is found in bark, root, leaves, and fruits of numerous medicinal plants, showed a wide range of biological activities such as anti-inflammatory, anticancer, antioxidant, cardioprotective, antiviral, and metabolic disorders [158–161]. In addition, UA functioned as a PPAR*α* agonist to regulate metabolic syndrome, liver diseases, respiratory dysfunction, and exaggerated inflammatory response in the animal and cell line experiments [158, 160, 162–164]. Likewise, UA improved cerebral ischemia/reperfusion injury, central nervous system (CNS) neural dysfunction, remyelination, multiple sclerosis (brain/central nervous system irregularities), and also airway inflammation of allergic asthma via promotion of PPAR*γ* signaling by its PPAR*γ* agonist potential in *in vivo* studies [159, 165, 166]. A recent study showed that UA (0-50 *μ*M) may exert antiskin cancer effects by promoting AMPK and PPAR*α* in Ca3/7 and MT1/2 premalignant and malignant skin cancer cell lines [166]. Also, ursolic acid in combination with artesunate suppressed hyperlipidemia and atherosclerosis due to increasing low density lipoprotein receptor (LDLR), apolipoprotein A-I (apoA-I), and PPAR*α*, as well as SREBP1 reduction in a hyperglycemic rabbit model [7]. Therefore, UA, a PPAR ligand and coactivator (Table 1), could play a role in management of multiple diseases, but future animal or clinical studies are needed to prove its promising properties related to PPARs.

### 4.17. Shogaol

6-Shogaol, the dehydrated form of 6-gingerols from dried *Zingiber officinale* (ginger) rhizomes, is a phenolic pungent compound which possesses numerous pharmacological properties, including anticancer, anti-inflammatory, and neuroprotective effects [167–169]. A number of studies reported that 6-shogaol acted as a PPAR*γ* agonist in its anti-inflammatory, antitumor, and neuroprotective effects (Table 1) [167–169]. These studies suggest that 6-shogaol may have a role as a novel PPAR*γ* agonist ligand to manage diseases such as inflammation, cancer, and neurodegeneration.

### 4.18. Oleic Acid

Oleic acid (OA) is the most abundant cis omega-9 monounsaturated fatty acid with 18 carbon atoms in olive oil, which exhibits antioxidant, cardioprotective, anti-inflammatory, antibacterial, and hepatoprotective effects [170, 171]. It has been reported that OA acts to enhanced PPAR*γ* to reduce TNF-*α*, IL-6, IL-1*β*, iNOS, and MMP-9 in monocytes or macrophages [171, 172]. Interestingly, OA repressed expression of PPAR*γ* and SIRT1 to protect coronary arteries in smooth muscle cells [170]. Also, OA boosted PPAR*δ* in HepG2 cells by provoking the G protein-coupled receptor 40-phospholipase C- (GPR40-PLC-) calcium pathway to regulate lipid metabolism and insulin sensitivity [172]. Thus, these results suggested that OA can function as a potential PPAR agonist (Table 1) and future work will be needed to investigate the relationship between PPARs and oleic acid on animal models and clinical trials.

### 4.19. Polyunsaturated Fatty Acid

Polyunsaturated fatty acids (PUFA) or essential fatty acids, known as n-3, n-6, or n-9, are found in fish and vegetable oils and have been shown to exert beneficial effects on human or animal health [173, 174]. Polyunsaturated fatty acids can act as PPAR signaling activators in the regulation of abnormalities in liver, cancer, cardiovascular, and inflammatory diseases (Table 1) [175–178]. In goats feeding with *α*-linolenic acid enhanced PPAR*α* in the liver [172]. While a number of studies have investigated PUFA effects on PPARs results were contradictory, and therefore more studies are warranted to determine their precise effects.

### 4.20. Other Phytochemicals

**Figure 2:**
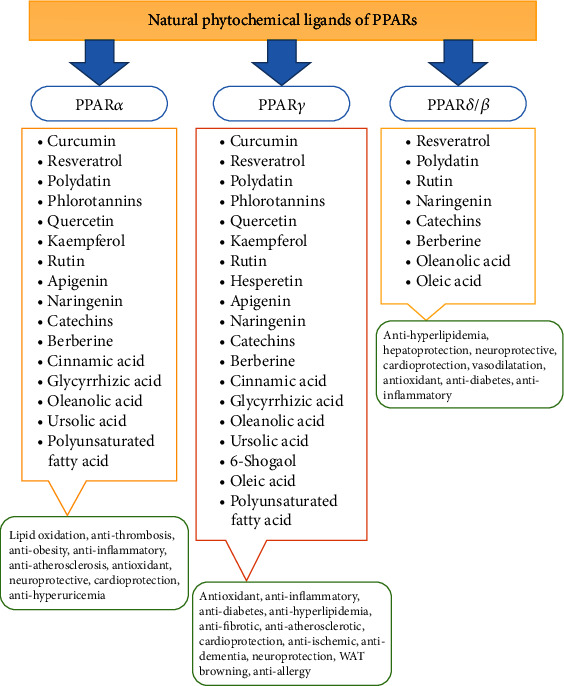
Phytochemical ligands of PPARs and their biological targets.

In addition to the compounds mentioned above, other natural phytochemicals showed potential PPARs ligand activity in research studies (Table 1). Terpenoids such as 1,8-cineole [7, 179], gingerol [7], cinnamaldehyde [180], carvacrol [181], zerumbone [182], oridonin [183], tanshinone IIA [184], pedunculoside [185], and lycopene and *β*-carotene [186] acted as dual PPARs activators for exhibiting antiatherosclerotic, antiadipogenic, anti-inflammatory, anticancer, hepatoprotective, and antihyperlipidemia effects. Interestingly, betulinic acid (a triterpenoid) had PPAR*γ* and PPAR*α* antagonist activity in 3T3-L1 cells to boost glucose uptake and osteogenesis, along with adipogenesis inhibition [187]. Also, fucosterol (a triterpenoid) [188], umbelliferone (a coumarin) [189], and chelerythrine (an alkaloid) [190] demonstrated PPAR*γ* activation in remediation of liver injury, liver fibrosis, and diabetes in animal models, respectively. The phytochemical ligands of PPARs and their biological targets are shown in (Figure 2).

## 5. Clinical Finding

Although numerous *in vitro* and *in vivo* studies demonstrated beneficial therapeutic effects of phytochemicals via their PPARs activation/suppression roles on a wide range of diseases (Table 1), there are few clinical studies on the impact of phytochemicals on PPARs and their implications in diseases. Limited clinical evidence for some phytochemicals associated with PPARs and disease remediation is available and is mainly on metabolic syndrome (Table 2). For polyunsaturated fatty acids (PUFAs), known to be PPAR ligands, most clinical trials have reported the role of eicosapentaenoic acid (EPA), docosahexaenoic acid (DHA), and *α*-linolenic acid (*n*-3 PUFAs) on PPARs activation/suppression to modulate disease [191–200]. Additionally, blood sampling or gene assay of subjects demonstrated single nucleotide polymorphisms (SNPs) associated with impacts of PPARs and PUFAs on metabolic syndrome [202, 204]. Moreover, curcumin and resveratrol increased expression of PPAR*γ* gene for regulation of metabolic syndrome and associated diabetes, coronary heart disease, and polycystic ovary syndrome [201–203]. In addition, administration of naringen into a diabetic 53-year-old African American female (a case study) showed that naringenin exerted its regulatory effects on insulin resistance and metabolic rate via activation of PPAR*α* and PPAR*γ*, leading to promotion of UCP1 and CPT1*β* [204]. In another study, effects of epigallocatechin gallate (EGCG) evaluated on Thai obese subjects (*n*=15) that reported consumption of 300 mg/day EGCG for 4 and 8 weeks did not affect expression of UCP1 and PPAR*γ* in browning white adipocytes, but interestingly EGCG reduced TG, blood pressure, and kisspeptin levels in these obese human subjects (Table 2) [205]. Given the sparsity of such clinical studies, the exact activation/suppression effects of phytochemicals on PPARs in diseases warrant more clinical trial investigations with larger sample size with attention to pharmacokinetic, dosage, frequency, and treatment duration protocols.

## 6. Limitations

There are some limitations to this review which are highlighted here. The most important limitation for therapeutic evaluation is the lack of sufficient clinical studies on the majority of PPAR natural agonists to date. In addition, there is insufficient evidence of safety or adverse side effects and possible drug interactions in oral administration of phytochemicals both in clinical and animal studies. Thus, further studies are needed to evaluate pharmacokinetic characteristics and bioavailability of phytochemicals as PPAR agonists. Likewise, as genetic polymorphisms in different individuals may modify the phytochemical effects on PPARs and their dosage and treatment regimes, there are only genetic polymorphic considerations of PUFAs in the available studies. While a wide range of natural phytochemicals have been suggested as candidate PPAR regulators from *in vitro* and *in vivo* studies, the greatest number of clinical trials have been performed on polyunsaturated fatty acids.

## 7. Conclusions

Overall, based on adjunct therapy with natural products in numerous diseases, this review has highlighted the interplay between phytochemicals and PPARs in multiple regulatory mechanisms of disease (Table 1). Here, we have focused on regulation by phytochemicals of disease abnormalities through PPAR-targeted molecular mechanisms, mainly from available *in vitro* and *in vivo* experimental models. However, clinical trials which were reported on the impact of phytochemicals in management of diseases via PPARs activation or suppression pathways are summarized in Table 2.

Based on the information presented in this review, it is noteworthy that phytochemicals have demonstrated promising potential, with acceptable safety, as agonists or antagonists of PPAR subtypes in several diseases associated with PPAR signaling cascades. In addition, phytochemicals not only can act as PPAR ligands but also they are able to impact on interactions with coactivators and corepressors in order for PPARs to target gene activation or suppression. Furthermore, phytochemicals also affect RXR activity and pre- and post-transcription regulators by inducing the obligatory heterodimer PPARs/RXR interaction, thereby instituting binding to PPRE and the consequent DNA binding site.

To conclude, it can be proposed that further studies warrant evaluation of more details of phytochemical formulations mentioned on their pharmacokinetic parameters, oral administration dosage, frequency, and absorption to enhance and expand clinical applications. As natural phytochemicals may represent favorable PPAR agonist/antagonist effects, it is expected that an understanding of phytochemical-mediated molecular mechanisms of PPAR-associated diseases will contribute to a safe approach to the therapeutic use of PPAR-targeted agents in the future.

## Figures and Tables

**Table 1:** Modulatory effects of phytochemicals on the PPAR family in diseases.

Phytochemical classification	Phytochemicals	General category of disease	Daily dose and treatment period	Experimental Model	Protective effect	Mechanism	Ref.
Polyphenols and simple phenols	Curcumin	Dyslipidemia	0.1% (w/w), oral feeding, 16 or 18 weeks	C57BL/6J obese mice	Antioxidant, anti-inflammatory, antihyperglycemic by reduction of lipid peroxidation, non-fasting blood glucose, oxidative stress, obesity, macrophage accumulation, and inflammation in eWAT	(+) PPAR*γ*, C/EBP*α* (-) Surf, TNF-*α*, IFN-*γ*, eIF2*α*, CD206, ALOX5, GPAT1, DGAT1, Pnpla2, F4/80, CD11c, MCP-1, IL-10, Rela, Leptin,NF-*κ*Bp65, SCD1	[9]
			100 mg/kg/day, feeding, 13 weeks	High-fat diet-induced obese mice	Antiobesity, antiapoptotic, autophagy regulation, anti-insulin resistance, antihepatic steatosis	(+) PPAR*α*, PPAR*γ*, AMPK (-) TG, Atg5, caspase 3, SREBP1	[10]
			10 *μ*M, 24 h treatment	Palmitic acid induced lipid droplet formation in AML12 cells	Autophagy regulation, antiapoptotic	(+) AMPK, LC3-Atg7, Bcl2/Bax (-) Lipid droplet formation	[10]
		Metabolic syndrome	80 mg/kg/BW, orally, 8 weeks	High fructose diet induced insulin resistance in rats	Antioxidant, antihyperglycemic	**(+)** PPAR*γ*, CAT, GSH, hexokinase, HDL, SOD **(-)** TBARS, lipid hydroperoxides, fasting blood glucose, TC, TG, LDL	[8]
			50 mg/kg/BW, oral gavage, 8 weeks	Male C57BL/6J obese mice	Antiobesity, antihyperglycemic	(+) PPAR*γ*, C/EBP*α*, HSL, ATGL, adipose triglyceride lipase (-) TG, TC, HDL-C, LDL-C, FFA, fasting blood glucose	[206]
			10, 20, and 35 *μ*M, treat 48 h	3T3-L1 adipocytes	Antihyperglycemic	(+) PPAR*γ*, PPAR*α*, C/EBP*α*, Glucose uptake (-) Glycerol release	[206]
	Curcumin+ Chromium		100 mg/kg/BW + 1mg/kg/BW, orally, 21 days	Streptozotocin-induced diabetic rats	Anti-inflammatory, antioxidant, and lipid lowering	(+) PPAR*γ*, GSH, adiponectin secretion (-) TC, TG, L-MDA, IL-6	[207]
			0.5%, 2% w/w, diet, 12 weeks	AApoAII female mice	Increasing hepatic lipid metabolism	(+) PPAR pathway, PPAR*α*, Apoa2 mRNA, CAT, CD36, Fabp, ApoE, HDL, amyloidosis, Scd-1 (-) TG	[208]
	Curcumin-mPEG454	Liver disease	50 and 100 mg/kg, IP, 16 weeks	HFD-fed C57BL/6J mice	Antiobesity, antihyperglycemic, anti-inflammatory, antisteatosis, antidiabetes, anti-insulin resistance, antiatherosclerotic	(+) CREB (-) PPAR*γ*, PPAR*γ*1, TG, hepatic steatosis, FFA, CD36	[25]
			10 *μ*M, 48 h treatment 80 mg/kg/BW, lavage, 5 weeks	Steatotic BRL cell, NAFLD rat	Antiobesity, hepatoprotective, antihyperglycemic, anti-insulin resistant	(+) PPAR*α* (-) DNA methylation level, TG, TC, ALT, AST, HOMA-IR, Serum glucose	[209]
			100, 200, and 300 mg/kg, gavage, 8 weeks	CCl4, olive oil-induced liver fibrosis rats	Antifibrotic, inducing hepatic stellate cell senescence, antiapoptotic	(+) PPAR*γ*, P53/*α*-SMA, Hmga1/*α*-SMA (-) HSC activation	[210]
			10, 20, and 40 *μ*M, 28 h treatment	HSC-T6 cell line	Antifibrotic, inducing hepatic stellate cell senescence, antiapoptotic	(+) P16, P21, Hmga1, Senescence-associated *β*-galactosidase-positive (-) *α*-SMA, *α*1(I)-procollagen, G0/G1 phase-related cyclins/CDKs	[210]
				100 and 200 mg/kg/day, IP, 8 weeks	HFD rat	Antihyperlipidemia, anti-inflammatory, fat degradation and suppression of lipogenesis, treat insulin resistance	(+) PPAR*α*, PPAR*γ*, CPT-1 (-) TC, TG, LDL, insulin resistance, Notch-1, Hes-1, NF-*κ*B, ACC, COX-2, TNF-*α*, SREBP-1c, FASN	[12]
			Cancer	2.5, 5, and 10 *μ*M, treat 24 h	TNBS-induced rat IEC-6 cell fibrosis	Antifibrotic	(+) PPAR*γ*, E-cadherin (-) Smad3, EMT, FN, CTGF, *α*-SMA	[211]
			Renal diseases	50 and 100 mg/kg, gastro gavage, 14 days	UUO-induced renal fibrosis mice	Antifibrotic	(+) PPAR*γ*, Smad2/3, ECM accumulation (-) FN, COL 1, PCNA, *α*-SMA, NRK-49F, cell cycle in G1 phase, cell proliferation	[30]
				10, 20, and 30 *μ*M, treat 48 h	NRK-49F cells			
				120 mg/kg, oral gavage, 5 days	Breast cancer-induced female Sprague-Dawley rats	Antifibrotic, anticancer, anti-inflammatory, antitumor	(+) PPAR*γ* (-) TNF-*α*, IL-6, IL-8,IL-10, BDNF	[212]
			Cardiovascular diseases	5 and 10 mg/kg, orally, 60 days	HFD-induced CMetS rats	Antioxidant, anti-inflammatory, Antifibrotic, collagen deposition, antihyperglycemic	(+) PPAR*γ*, HDL-C, GSH, myocardial marker (-) TNF-a, IL-6, NF-*κ*B, hs-CRP, glucose, insulin, insulin resistant, TC, TG, LDL-C, TBARS	[31]
				20 *μ*M, treat 1 h	Angiotensin II-induced inflammatory rat VSMCs	Anti-inflammatory, antioxidant, antiproliferative	(+) PPAR*γ* (-) IL-6, TNF-*α*, NO, iNOS, p47phox,iROS,	[33]
				150 mg/kg/BW, Intragastric, 4 weeks	Rat myocardial infarction	Anti-ischemic, anti-inflammatory, antiapoptotic, antioxidant, antinecrotic	(+) PPAR*γ*, Bcl-2 (-) NF-*κ*Bp65	[32]
				100 mg/kg/day, orally, 12 weeks	Spontaneously hypertensive rats	Antihypertension, antifibrotic		[213]
				5, 10, and 20 *μ*M, treat 1 h	Rat cardiac fibroblasts		(+) PPAR*γ* (-) Ang II, CTGF, PAI-1, ECM production, TGF-*β*/Smad2/3, systolic blood pressure, collagen III, fibronectin	
				100 mg/kg/day, orally, 6 weeks	Diabetic ratcardiomyopathy	Cardioprotection, antioxidant, anti-inflammatory, regulate lipid metabolism, prevent heart failure	(+) PPAR*γ*, TAC, GSH, HDL-C (-) CaMKII/NF-*κ*B/TGF-*β*1, lipid peroxidation, Blood glucose level, CK-MB, troponin I, MDA, TNF-*α*, NF-*κ*B, IL-6, TG, TC	[214]
				0.02% w/w, diet, 18 weeks	LDLR^−/−^ mice	antiatherogenic, lipid-lowering, antihyperglycemic, immunity	(+) PPAR*α*, LXR*α*, HDL-C, Apo A-I (-) Cholesterol, TG, LDL-C, Apo B, CETP, HMG-CoA reductase, ACAT1, ACAT2, Cfd, CRP, ICAM-1, VCAM-1	[11]
			Brain and nervous systemdiseases	10*μ*M, treat 1 h	OGD/R-induced injury rat cortical neuron cells	Anti-ischemic, antiapoptotic, neuroprotection, antioxidant	(+) PPAR*γ*, Bcl-2, Cyt c, AIF, JC-1, (-) LDH, I*κ*B-*α*,NF-*κ*Bp65, NF-*κ*B, NO, Bax, caspase3, ROS, IKK, DCFDA	[215]
				20 mg/kg/d, P.O., 14 days	STZ-induced Swiss albino mice dementia	Antidementia, antioxidant	(+) PPAR*γ*, GSH, (-) AChE, TBARS	[216]
				1 or 5 *μ*M, treat 24 h	Rat OPs-myelin diseases	Protect against demyelination, anti-inflammatory	(+) PPAR*γ*, PGC1-*α*,COX1, ERK1/2, Caspase3, MBP, O_1_,O_4_, (-) OP metabolic, TNF-*α*	[217]
				150 mg/kg, IP, 4 weeks	APPswe/PS1*Δ*9 transgenic mice	Anti-Alzheimer, anti-inflammatory, improved memory function, neuroprotection,	(+) PPAR*γ*, ChAT, Ach (-)NF-*κ*B, LDH, TNF-*α*, IL-1*β*, COX-2, NO,GFAP, Mac-1,I*κ*B-*α*, NF-*κ*B p65	[37]
				40 mg/kg, 4 weeks	Primary cultured mouse astrocytes	Anti-Alzheimer, anti-inflammatory, neuroprotection	(+) PPAR*γ* (-) COX-2, amyloid-*β*, astroglia	[34]
				200 mg/kg, IP, 3 days	Rat middle cerebral artery occlusion	Anti-ischemic, anti-inflammatory, neuroprotection, decreased infarct volume	(+) PPAR*γ*, PPAR*γ*-PPRE, (-) IL-1*β*, I*κ*B-*α*, TNF-*α*, NF-*κ*B p65, PGE2, NO, iNOS, COX-2	[35]
				10*μ*M, treat 24h	APOE4-induced neurological SH-SY5Y cell damage	Anti-inflammatory, neuroprotection	(+) PPAR*γ* (-) TNF-*α*, IL-1*β*, NO, COX-2, iNOS, NF-*κ*B p65	[36]
			Respiratory	0-50 *μ*M, treat 48h	TGF-*β*1-induced human lung CCD-19Lu fibroblasts	Antifibrotic, anti-inflammatory,	(+) PPAR*γ*, CatB, CatL, stefin B, (-) ERK, *α*-SMA, TGF-*β*1, collagen I, Col1a1, Col1a2, cystatin C,	[20]
			Immunity	10, 30, and 100 *μ*M, treat 48 h	Preeclamptic PBMC	Anti-inflammatory	(+) PPAR*γ* (-) IL-1*α*, IL-6, TNF-*α*, NF-kB p50	[38]
				100 *μ*g, IP, 12 or 13 days	EAE C57BL/6 mice	Immunity, anti-inflammatory	(+) PPAR*γ*, IL-10, CD4^+^CD25^+-^Foxp3^+^ Treg, CD4^+^ Thelper (-) IFN*γ*, IL-17, IL-12, IL-23	[218]
				0, 2.5, 5, 10, and 25 *μ*M,	Spleen cells of EAE mice	Immunity, antimalarial		
				10 *μ*M, treat 24h	Human THP-1 monocytes		(+) PPAR*γ*, CD36, monocyte ROS, Nrf2	[219]
		Resveratrol	Dyslipidemia Metabolic syndrome	1, 5, and 10 *μ*M, treat 2 h	Human monocytic leukemia THP-1 cells	Antiatherosclerotic, lipid-lowering, antioxidant	(+) PPAR*γ*, ABCA1, ABCG1, (-) SR-A, RAGE, foam cell formation, cholesterol accumulation	[40]
				10 mg/kg/ twice a day, orally, 24 weeks	Male ApoE^−/−^ atherosclerotic mice	Antiatherosclerotic, lipid-lowering, antioxidant, attenuate changes in carbohydrate metabolism and amino acid metabolism	(+) PPAR*α*, PPAR*γ*,ABCA1, ABCG1, (-) monoglyceride accumulation, cholesterol accumulation, TC, CE, neutral lipids	[41, 206]
				1.5 *μ*g/ml, treat 24 h	Mice RAW264.7 macrophages			
				20 mg/kg/day, gavage, 2 weeks	HFD-induced obese/diabetes mice HUVECs	Antioxidant, antidiabetes, antiobesity, improved endothelium-dependent relaxations	(+) PPAR*δ*, SIRT1,eNOS, Akt, PPRE luciferase (-) ROS, BW, HW, subcutaneous fat weight	[220]
				20 *μ*M, treat 24 h				
				50 *μ*M, treat 24h	C2C12 myoblast hypoxic cell line	Antioxidative metabolism, anti-insulin sensitivity	(+) PPAR*α*, PPAR*γ*, SIRT1, RXR-*α*, UCP2 (-) Lipid peroxidation, ROS	[221]
				100 mg/kg/day, gavage, 12 weeks	Catch up growth rat	Anti-inflammatory, antiobesity, fat lowering, balance between lipid production and storage, ameliorating insulin sensitivity	(+) SIRT1, FSP27, GIR60–120, adipose tissues glucose, adiponectin (-) PPAR*γ*, FINS, TNF-*α*	[222]
		Resveratrol+ quercetin		10 or 50 mg/kg/day, orally	WAT from MetS rats	Improving lipid metabolism, antiobesity, antidyslipidemia, antioxidant, anti-inflammatory Antioxidant, protect lifestyle-related diseases	(+) PPAR*α*, MUFA, PUFA, UCP2 (-)UCP3, dihomo-*γ*-linoleic, SFA	[223]
		Resveratrol and Vaticanol C (resveratrol tetramer)		4weeks 0.04%, feeding, 8weeks	HFD mice		(+) PPAR*α*,PPAR*β*/*δ*, cyp4a10, cyp4a14, FABP1, UCP3, PDK4 (-) ROS	[224]
			Liver disease	200 and 400 mg/kg/day, liquid diet feeding, 2 weeks	Alcoholic fatty liver mice	Reduced lipid synthesis, increased rates of fatty acid oxidation, prevented alcoholic liver steatosis	(+) SIRT1, AMPK, PGC-1*α*, AdipoR1/R2, circulating adiponectin, FOXO1, CPT1a, MCAD, AOX (-) SREBP-1, PPAR-*γ*, TNF-*α*, SCD1, FAS, GPAT1, ACC*α*, ME	[225]
				100 mg/kg/day, gavage, 8 weeks	HFD-induced NAFLD in rats	Antioxidant, improved lipid metabolism and mitochondrial respiratory chain activity	(+) PPAR*α*,AMPK,PKA,CPT-1,SOD, CAT, T-AOC, complex I, complex IV (-) SREBP-1c, FAS, MDA, ALT, AST, LDL-C, TC, TG	[226]
			Cancer	30 or 50 *μ*M, treat 24,48h	Human colon carcinoma cell lines SW480, HCT116, Caco-2, SW620	Antiapoptotic, cancer cell cycle arrest effect, antitumor, accumulation of tumor cells in the S phase	(+) PPAR*γ*, caspase3, pcDNA3 (-) Cell survival	[227]
				5 g/kg, diet, 5 weeks	Ovariectomized female C57BL/6 mice	Antiobesity, anticancer, anti-inflammatory, antimammary adipocyte hypertrophy, prevented macrophage infiltration, CLS prevalence, and M-Wnt murine mammary tumor size	(+) PPAR*γ* (-) IFN-*γ*, IL-1*β*, IL-6, COX-2, MCP-1, TNF-*α*, Wnt/*β*-Catenin	[228]
			Renal diseases	40 mg/kg, orally, 6 months	Male C57BL/6 mice	Antioxidant, anti-inflammatory, improved renal function, antifibrotic, prevents diabetic nephropathy, prevent lipotoxicity	(+) PPAR*α*, CrCl, Bcl-2, Nrf2,HO-1, NQO-1,SIRT1, AMPK, PGC-1*α*, ERR-1*α*, SOD1, SOD2,COX I/COX IV (-) HbA1c, albumin, SCr, eGFR, Col IV, TGF-*β*1, F4/80, Bax,8-OH-dG, urinary isoprostane,Lys-PGC-1*α*, SREBP1,PI3K,Akt, FOXO3a	[229, 230]
				50 *μ*M, treat 24h	HK2 cell			
				20 mg/kg/day, gavage, 12 weeks	C57BLKS/J *db/db* mice			
				400 mg/kg/day orally, 12 weeks	HFD-C57BL/6J mice	Antilipotoxicity, antiobesity, anti-inflammatory, antifibrotic	(+) PPAR*α*, AMPK, lipolysis (-) TG, lipid, 4-HNE,TNF-*α*, IL-6, iNOS, BUN, albumin, eGFR,	[231]
			Cardiovascular diseases	100 mg/kg/day, IP, 6 weeks	*db/db* C57BL/6 mice	Antihyperlipidemic, antihyperglycemic, cardioprotection, improved cardiac function	(+) PPAR*α*/*γ*, SIRT1,mtDNA, FS (-) PGC-1*α*, TG, glucose	[232]
				10 and 25 *μ*M, treat 18 h	*THP-1 monocytes, HAEC*	Antiatherosclerosis, antioxidant, lipid lowering	(+) PPAR*γ*, cholesterol efflux, ABCA1, LXR-*α*, 27-OH, SR-B1, CD36, (-) Foam cell formation, oxLDL, lipid accumulation	[233]
				50 mg/kg/day, IP, 5 days 50 *μ*M, treat 24	Hypertrophic neonatal rats NCMs	Cardioprotection, anti-inflammatory, lipid lowering, antioxidant	(+) PPAR*α*, SIRT1, PDK4, mCPT-I, MCAD (-) NF-kB, PGC-1*α*, AdGFP, ANF, *α*-SKA, MCP-1	[234]
				(0.01, 0.1, 1, 5, and 10) *μ*M, treat 48 h	EPCs	Antioxidant, enhanced re-endothelialization, inhibited EPC senescence, repaired endothelium	(+) PPAR*γ*, h-TERT, HO-1, NO (-) NADPH, SIRT1, ROS	[235]
		trans (t)-resveratrol		15 mg/kg/day, IV, 1 month	Ang-II-induced rat vascular inflammation Leukocyte-HUVECs MCAO mice	Cardioprotection, inhibition of AT1 receptor, anti-inflammatory	(+)PPAR*γ* (-) MCP-1, MIP-1*α*, CAM, leukocyte, p38 MAPK,NF-*κ*B p65,CD11b, CINC/ KC, VCAM-1, P-selectin,IL-8, ICAM-1, RANTES	[236]
		Malibatol A (a resveratrol oligomer)	Brain and nervous system diseases	1–10 *μ*M, treat 1h 20 mg/kg, injection, 15 min after the onset reperfusion		Antioxidant, anti-ischemic, anti-inflammatory, immunity, neuroprotecion	(+) PPAR*γ*, IL-10, TGF-*β*, CD206, YM-1 (-) TNF-*α*, IL-1*β*, iNOS, IL-6, CD16, CD32, CD86	[237]
		Polydatin	Dyslipidemia	8.9 *μ*g/mL, treat,48 h	Peritoneal macrophages of ApoE^−/−^mice	Anti-atherosclerotic, anti-inflammatory, prevented foam cells formation in peritoneal macrophages	(+) PPAR*γ*, ABCA1 (-) TC, free cholesterol, cholesterol ester, TNF-*α*, IL-1*β*, CD36,	[50]
			Metabolic syndrome	100 mg/kg/d, oral, 4 weeks	HFD mice	Antiobesity, anti-inflammation, body weight loss	(+) PPAR*γ*, HDL, leptin (-) TC, adipose cell sizes, fat mass, TG, LDL, MCP-1,TNF-*α*,	[238]
				100 mg/kg, PI, 12 weeks	HFD-ApoE^−/−^ mice	Antiatherosclerotic, anti-inflammatory, reduced atherosclerotic plaques in aortic arch and sinus, down-regulation of cholesterol metabolism related gene transcription, losing blood lipids.	(+) HDL, T-SOD (-) TC, TG, LDL, ALT, AST, MDA, SREBP1, Fasn, HmgcR, PBEF, PPAR*γ*, cholesterol uptake, IL-6, TNF-*α*, hs-CRP,	[239]
				50, 100, and 200 *μ*g/mL, treat, 2h	RAW 264.7 cells			
				1, 3, and 10 *μ*mol/L, treat, 6 h	Rats aortas	Antihyperglycemia, improved the histological damage to endothelial cells, restored the relaxation under acetylcholine	(+) eNOS, NO, PPAR*β* (-) iNOS	[52]
			Liver disease	50 and100 mg/kg/d, intragastric,4 weeks	STZ-HFD mice(diabetic hepatopathy mice)	Hepatoprotection, antidiabetes, anti-inflammatory, lipid lowering	(+) PPAR*β*, PPAR*α* (-) TNF-*α*, IL-1*β*, TC, TG, ALT, AST, ALP, FBG, NF-*κ*B p65, iNOS, COX-2	[53]
				7.5, 15, and 30 mg/kg, intragastric,7 weeks	Fructose-associated liver inflammation and lipid deposition rats	Antioxidant, anti-inflammatory, antihyperlipidemic	(+) PPAR*α*, Keap1/Nrf2, miR-200a, CPT-1, GST, HO-1, NQO1, (-) ROS, TG, TC, TXNIP, NLR, NLRP3, SREBP-1, SCD-1, TNF-*α*, IL-1*β*, ASC, Caspase 1,	[54]
				10, 20, and 40 *μ*M, treat, 48 h	Buffalo rat liver cells, HepG2			
			Cardiovascular diseases	200 *μ*M/kg, gavage, 6 weeks	Rats	Increased arterial pressure and heart rate, decreased QRS interval and slightly reduce ST and QT intervals, attenuate myocardial pathological damage, improving energy metabolism	(+) AMPK-*α*2, PPAR-*α*, SOD, GSH-Px, Na^+^K^+^-ATPase, Ca^2+^Mg^2+^-ATPase, PCr, ATP, ADP, TAN, PCr/ATP (-) MDA, FFA	[240]
			Brain and nervous system diseases	20 *μ*M, treat, 24h	Ischemic rat brain microvascular Endothelial cells	Anti-inflammatory, anti-ischemic, antiapoptotic	(+) PPAR*γ*, MALAT1,CREB, PGC-1*α*, C/EBP*β* (-) LDH, TNF-*α*, IL-6, COX-2, Claudin-5, Occludin, ZO-1, ICAM-1, VCAM-1, MCP-1	[241]
			Respiratory	50, 100, and 200 mg/kg/d, intragastric, 28 days	Bleomycin-induced pulmonary fibrosis in SPF male mice	Antipulmonary fibrosis, anti-inflammatory	(+) PPAR-r (-) NF-kB, cytokines	[242]
				50 mg/kg, oral, 8 weeks	aPM2.5-induced rat lung injury	Anti-inflammatory, antioxidant	(+) PPAR*γ*, GSH-Px, Nrf-2 (-) ROS, MDA, ICAM-1, MCP-1, IL-6	[243]
		Phlorotannins	Metabolic syndrome	12.5, 25, and 50 *μ*M, treat,8 days	3T3-L1 adipocytes cells	Suppressed adipocyte-specific genes and lipid formation, antiadipogenic, reduced lipid accumulation, antiobesity	(-) PPAR*γ*, C/EBP*α*, lipid content	[55]
		Phloroglucinol of *Potentilla longifolia*		10, 20, 40, and 80 *μ*M, treat, 96 h	3T3-L1 cells	Inhibited lipid accumulation	(+) AMPK, ACC (-) SREBP1c, FAS, SCD1, PPAR*γ*, C/EBP*α*, TG	[58]
		*Ecklonia stolonifera* extract	Liver diseases	50, 100, and 200 mg/kg/day, gavage, 4 weeks	Ethanol-induced fatty liver Rat	Antioxidant, hepatoprotective, lipid lowering, anti-inflammatory	(+) PPAR*α*, CPT-1 (-) TG, SREBP-1, TC, ALT, AST, MDA, FFA	[244]
		Quercetin	Metabolic syndrome	0.19 and 0.95 mg/Kg/day, orally, 4 weeks	WAT from MetS rat	Reduced adipogenesis in preadipocytes, body weight, central adiposity, insulin concentration, and systolic arterial pressure	(+) PPAR*γ*, HDL-C, SIRT 1, SIRT 2,PUFA (-) TC, TG, SIRT 3, leptin, MUFAs, NEFAs	[68]
				0.05%, gavage, 9 weeks 10 *μ*M, treat 40–42 h	WAT of HFD-fed obese mice 3T3-L1 adipocytes	Antiobesity, thermogenic activator, induced browning of WAT, improved metabolic complication	(+) PPAR*γ*, PGC1*α*, Tfam, Tmem26, Cidea, Prdm16, Nrf1, UCP1, PKA/AMPK, *β3AR* (-) -	[69]
				5, 10, and 50 *μ*M, treat, 24 h	OP9 cells	Prevented adipogenesis, regulated lipolysis enzymes, antiobesity, antiadipogenic	(+) ATGL, HSL (-) C/EBP*α*, PPAR*γ*, SREBP-1c, lipid accumulation, FAS, LPL, aP2	[245]
				0.3, 1.5, 3, 15, and 30 *μ*M, treat,8 h	THP-1 cells	Antiatherogenic, antihyperlipidemic	(+) PPAR*γ*, LXR*α*, ABCA1, HDL,apoA1 (-) -	[70]
				25, 50,100, and 200 *μ*M, treat, 4, 8,16,32 h	THP-1 cells	Decreased formation of foam cell derived, increased cholesterol efflux from macrophages	(+) PPAR*γ*-LXR*α*, ABCA1, PPRE-luc reporter (-) TC, lipid droplets	[246]
				5, 10, and 30 *μ*M, treat,6 days	Male F344 rat primary mSCs	Suppressed lipid accumulation, and mSC adipogenesis	(+) - (-) PPAR*γ*, FABP4, TG	[247]
				0, 0.2, 0.4, and 0.6 g/kg, feeding, 42 days	AA broilers	Decreased abdominal fat, improved lipid metabolism	(+) PPAR*α*, PI3K, AMPK*α*1, AMPK*α*2, AMPK*β*2, LKB1, PKB, AMPK*γ*, CPT1, (-) PPAR*γ*, SREBP1, ACC, HMGR	[248]
		Quercetin-3-O-*β*-D-glucuronide		25 and 50 mg/kg/day, gavage, 8 weeks	HFD-male SD rats	Reduced bodyweight, liver weight, liver index, fat overload, lipid accumulation and dyslipidemia, anti-inflammatory, antiapoptotic, hepatoprotective	(+) PPAR*α*, HDL, CPT1, MCAD (-) TG, SREBP-1c, TNF-*α*, IL-6, ALT, AST, LDL, TC, FAS	[249]
				1%, feeding, 16 weeks	HFD mice	Antiobesity, increased WAT browning, increased lipolysis,	(+) PPAR*γ*, UCP1, PGC1*α*, BAT, ACSL4, ACOT11, ADRB3 (-) TC, p38	[250]
				5, 10, and 20 mg/L, treat, 24, 48, and 72 h	AA broiler hepatocytes	Enhanced lipid transportation and*β*-oxidation of FA, reducing lipid deposition	(+) PPAR*α*, ACSL, ApoA1,FABP, (-) ApoC3, VLDL, TG	[251]
		Isorhamnetin		100 mg/kg/d, gavage, 2 weeks 1% (W/W), diet, 4weeks	*ob/ob* mice HFD-C57/BL6 mice	Reduced body weight and fat, ameliorated insulin resistance, alleviated hepatic steatosis	(+) MRC II, III, IV, V (-) PPAR*γ*, aP2, CD36, LPL, ACC, SREBP -1c, aP2, CD36, SCD1, AUC, FFA, Leptin, Insulin level, Blood glucose, TG, TC	[252]
				12.5, 25, and 50 *μ*M	3T3-L1 preadipocyte HFD rat	Antioxidant, augmented adiponectin expression, increased the concentration of circulating Adiponectin	(+) Plasma adiponectin, FFA (-) PPAR*γ*, HOMA, 8-iso-PGF2*α*, TG	[253]
				25 mg/kg/BW, gavage, 4 weeks				
		Q, Q2		1, 5, 10, and 25 *μΜ*, treat, 7 days	Mouse 3T3-L1 cells	Suppressed lipid accumulation, adipocyte area, antiobesity, attenuated adipogenesis	(+) PPAR*γ*, cEBP*α*, HDL, adiponectin (-) BW, BMI, abdominal fat, heart weight, cardiac somatic index, glycemia, insulin, HOMA, TC, TG, LDL	[254]
				0.26 mg/kg, orally, 12 weeks	HFD rat			
				25 mg/kg/day, IP, 4 weeks	Male C57BL/6 mice	Antidiabetic, antiobesity, reduced hyperlipidemia, hyperglycemia, adipogenesis	(+) GLUT4, Akt, AMPK, insulin sensitivity, glucose tolerance (-) PPAR*γ*, FABP4, cEBP*α*, BW, TC, TG, glucose, LDL	[255]
				1, 10, and 50 *μ*M, treat,48 h	3T3-L1 adipocytes			
		Q derivatives+1% catechin	Liver disease	10^−5^ M, diet, 5 days	HFD-C57BL/6N mice	Antioxidant, antiobesity, improved lipid and glucose metabolism, enhanced *β*-oxidation	(+) PPAR*α*, Ehhadh, GK (-) gp91phox, FAS, GPAT, L-PK, G6Pase	[256]
				6.25, 12.5, and 25 *μ*M, treat, 24 h	3T3-L1,RAW 264.7 cells	Anti-inflammatory, antiobesity, inhibited adipogenesis and lipogenesis, inhibited lipid accumulation and body weight	(+) IL-10, adiponectin, insulin sensitivity, HDL (-) PPAR*γ*, mTOR, PI3K, Akt, p70S6K, C/EBP*β*, C/EBP*α*, FABP4, DGAT1, LPAAT*θ*, Lipin1, ERK,JNK,P38MAPK, TNF-*α*, IL-1*β*, IL-6, AP-1, MCP-1, NF-*κ*B, TG, LDL	[257]
				25,50,100 mg/kg, oral, 10 weeks	HFD mice			
				10, 20 mg/kg/BW, gavage, 6weeks	Hepatocarcinogenesis rats	Antioxidant, prevented early stages of liver cancer and neoplastic foci, induced apoptosis	(+) CAT, SOD, Caspase3, p53, Bax/Bcl-2, cytochrome c (-) PPAR*γ*, PPAR*α*, TBARS, cyclin D1, cyclin A, cyclin B1, cdk1	[258]
				50 and 100 mg/kg, gavage, 4 weeks 20 *μ*M, treat, 24h	NAFLD rat RHPCs	Antioxidant, anti-inflammatory, decreased lipid accumulation, antiapoptotic	(+) PPAR*α*, MMP, SOD, TAC, Nuclear Nrf2, (-) NOX, TXNIP, ROS, H_2_O_2_, MDA, iNOS, XO, O_2_^•−^, NLRP3, ASC, Caspase 1, IL-1*β*, IL-18, JAK2, STAT3, SOCS3, SREBP1, SCD1	[259]
				50 *μ*M, treat, 48h	Oleic acid-induced lipid accumulation Huh7.5 cells	Decreased intracellular lipids and LD size, downregulate hepatic lipogenesis, upregulate lipolysis, reduced steatosis	(+) PPAR*α* (-) TG, SREBP-1c, PPAR*γ*, ACAT1, apoE, apoB	[260]
				0.08% in the AIN-93G diet, 10 weeks	*ob/ob* mice	anti-inflammatory, antioxidant, controlled hypercholesterolemia, alleviated hepatic steatosis, improved liver function, alleviation of insulin resistance, enhanced fatty acid oxidation, suppressed lipogenesis,	(+) PPAR*α*, AMPK, adiponectin, (-) FFA, ALT, SREBP-1c, PPAR*γ*, TNF-*α*, MCP-1, cholesterol, TC, HOMA-IR, TG	[261]
				50 mg/kg, oral, 6 weeks	High-fat high-sucrose-rats	hypolipidemic action, modulated metabolic markers	(+) G6PDH, (-) PPAR*γ*, TG, TC, lipase, G3PDH, Adipose, hepatic tissue	[262]
		QP	Cancer	100 mg/kg, gavage, once a week/12 weeks 2 and 10 *μ*M, treat, 24 h	Mongolian gerbils Human A549 lung cancer cells	Suppressed cell invasion and migration, anticancer, cell cycle arrest at the G2/M phase, antiproliferative	(+) PPAR*γ*, nm23-H1, TIMP-2, PTEN (-) MMPs-2, cdk1, cyclin B, p-Akt	[263, 264]
		Isorhamnetin		10, 25, and 50 *μ*M, treat, 24-72 h	Human AGS cell line	Anticancer, anti-inflammatory, antiproliferative, pro-apoptotic	(+) PPAR*γ*, PPAR*β*, caspase 3, caspase 9 (-) Bcl2, Cyclin D1, Bcl-xL,CD31	[265]
			Renal diseases	50 and 100 mg/kg/day, gavage, 4 weeks	Cd-induced nephrotoxicity rats	Inhibited lipid accumulation, antihyperuricemic, antidyslipidemic, nephroprotective	(+) PPAR*α*, Renal XDH, FEUA, CPT1, AMPK, OCTN2, (-) Urine RBP, Urine *β*-MG, Urine ALB, Serum UA, uric acid, Renal XO, Renal RST, Renal OAT1, TG, VLDL, SREBP-1,PGC-1*β*,	[266]
			Cardiovascular diseases	50 mg/kg/d, gavage, 8 weeks	C57BL/6 mice	Antiatherosclerotic, increased lipid droplets and lipid uptake, antioxidant	(+) PPAR*γ*, SR-BI, Dil-HDL, LXR*α*, SLU, HDL-C (-) Lipid accumulation, oxLDL level	[60]
				15 *μ*M, treat, 6, 12, 24 h	HepG2 cells			
				12.5 mg/kg, oral gavage, 12 weeks	HFD-apoE^−/−^ mice	Antiatherosclerotic, anti-inflammatory, antioxidant, reduced the atherosclerotic plaque area, increased the collagen fibers in atherosclerotic plaques, improved hepatocyte microstructure	(+) PPAR*γ*, LXR*α*, ABcA1, HDL, IL-10 (-) CD36, PcSK9, TC, TG, LDL, oxLDL, TNF-*α*, IL-6, lipid accumulation, FC	[63]
				50, 100 mg/kg, gavage, 1 week	Hypertensive rats	Antioxidant, anti-inflammatory, antiapoptotic, increased cardiac and renal antioxidant enzymes, cardioprotective effects	(+) PPAR*γ*, Hsp70, ERK, GSH, GPx, SOD, GST, (-) SBP,DBP, MAP, AST, PT, NPT, Cyt C, p38, MDA, AOPP, H_2_O_2_,	[64]
				5 and 10 mg/kg, gavage, 12 weeks 100 *μ*g/ml, treat, 24 h	Spontaneously hypertensive rats Angiotensin II-induced H9C2 cells	Reduced hypertrophic surface area, reduced blood pressure and left ventricular weight	(+) PPAR*γ*, LVIDd (-) AP-1, ANP, BNP,SBP, IVSd, LVPWd, c-fos, c-jun, CVF	[65]
				250 mg/kg/d, gavage,10 days 40 *μ*M, treat, 24 h	MI-C57/BL6-mice Hypoxia H9C2 cell lines	Anti-ischemic, increased ejection fraction and fractional shortening, antioxidant, anti-inflammatory, antiapoptotic	(+) PPAR*γ*, SOD, GSH-PX, (-) CK-MB, AST, cTnT, LDH, iNOS, MDA, caspase-3, NF-*κ*B p65, I*κ*B*α*	[66]
				0.5% w/w, gavage, 4 weeks	Cardiac dysfunction in hyperglycemic rats	Antihyperlipidemia, improved cardiac function, inhibited cardiac cholesterol and heart weight, antioxidant	(+) HDL, Nrf2, HO-1,SOD, CAT, GSH, ATP levels, PGC-1*α*, (-) PPAR*γ*, TC, TG, glucose, VLDL, LDL, TBARS, UCP2	[267]
		Kaempferol	Metabolic syndrome Dyslipidemia	5-20 *μ*g/mL, treat, 24 h 40 and 80 *μ*M, treat, 72 h 60 *μ*M, treat, 21 days	3T3-L1 adipocytes	Increased adiponectin, antiadipogenic, suppressed lipid accumulation, antiobesity, anti-inflammatory, antioxidant	(+) Free glycerol release, TNF-*α*, Pnpla2, Lipe (-) PPAR*γ*, C/EBP-*α*, FAS, leptin, SREBP-1c, adipsin, LPL, aP2, Dgat2, Agpat2, Scd1, Lsr, Cel, GLUT4, CD36, LPIN1, Resistin, LXR*α*, LXR*β*, C/EBP-*β*	[76, 78, 80]
				1, 10, or 25 *μ*M, treat, 48 h	hMSCs	Antiadipogenic, delipidating effects, reducing lipogenesis	(+) Atgl (-) PPAR*γ*, C/EBP-*β*, TG	[81]
				0.15% dietary, 92 days	HFD-obese C57BL/6J mice	Antiobesity, antidiabetic, reduced adipose tissue accumulation, increased lipid metabolism	(+) Insulin resistance, (-) PPAR*γ*, TNF-*α*, fasting blood glucose, HbA1c, SREBP-1c, body weight	[77]
				20 *μ*M, treat, 24 h	Hek-293 cells transfected with luciferase reporter constructs	Antioxidant, longevity-associated transcription factors	(+) PPAR*γ*, Nrf2, FoxO (-) ROS	[82]
				10 or 20 *μ*M, treat, 24 h 150 mg/kg/d, orally, 10 weeks	HepG2, THP-1, and Caco2 ApoE-deficient C57BL/6J mice	Promoted lipid metabolism, induced hepatic autophagy, motivated macrophage cholesterol efflux, stimulated fatty acid oxidation and uptake, blocked SREBP1 translocation to nucleus	(+) PPAR*α*, PPAR*δ*, Insig-2a, 2-NBD-cholesterol efflux, Lxr_*β*_, ABCA1, ABCG5, ABCG8, Apoe, ACADL, CPT-1a, ACOX-1, APOC3, fatty acid uptake, LC3-II, LAMP-1, LAMP-2, APG-7 (-) Akt, TG, SREBP-1, NCP1L1, GSK-3, S6K1	[79]
		Rutin	Metabolic syndrome	50, 100, and 200 mg/kg, IP, 5 weeks	db/db mice	Lipid lowering, reduced glucose	(+) PPAR*γ*, LPL (-) Blood glucose, TG	[83]
				3, 10, 30, and 100 *μ*M, treat, every 2 days for 8 days	Mouse 3T3-L1 adipocytes	Increased lipid accumulation, stimulated adiponectin secretion, adipogenesis	(+) PPAR*γ*, C/EBP*α*, aP2, adiponectin secretion, adiponectin (-) -	[84]
				100 mg in 100 g HFD, 16 weeks	HFD-C57BL/6J mice	Restored glucose and insulin tolerance, reduced ER stress markers, adiponectin	(+) PPAR*γ*, DsbA-L, p-JNK, Akt (-) Adiponectin,GRP78, insulin	[85]
				0.1%, diet, 16 weeks	HFD rat	Improved mitochondrial loss, increased functional capacity in skeletal muscle, decreased total weight, lipid-lowering, decreased adipogenesis, antiobesity	(+) HDL, AMPK, mtDNA,NRF1, Tfam, PGC-1*α*, SIRT1, CPT1 (-) PPAR*γ*, SREBP-1c, aP2, atherogenic index, TG, LDL	[86]
				2, 10, and 50 *μ*M, treat, 48 h	Murine 3T3-L1 cells	Antiadipogenic, suppressed lipid accumulation	(+) AMPK (-) PPAR*γ*, C/EBP*α*, Lipin1, FAS, LPL, aP2	[87]
		Rutin and quercetin	Renal diseases	50 and 100 mg/kg, gavage	Fructose-fed rats	Antihyperuricemia, antidyslipidemia, restored renal dysfunction, antioxidant, anti-inflammatory, lipid accumulation	(+) rPPAR*α*, rCPT1, rOCTN2, L-carnitine, rJAK2, rIR, rAkt, rIRS1(Tyr), rERK1/2 (-) rNLRP3, rASC, rCaspase-1, uric acid, TG, TC,VLDL, creatinine, BUN, insulin, leptin, rOb_L_, p-rOb_L_, p-rSTAT3, IL-1*β*, IL-6, IL-18, rTNF-*α*, rSOCS3, p-rOb-RL (Tyr1138), rIRS1(Ser)	[88]
			Brain and nervous system diseases	30 mg/kg, oral, 14 days	Cisplatin induces neurotoxic rats	Neuroprotective, antioxidant	(+) PPAR*δ*, PON-1, PON-3, GPX, glutathione (-) PON-2, TBARS	[89]
			Immunity	11.5 mg/kg bw, oral, 4 weeks	Ovalbumin-induced sensitive Balb/c mice	Immunity, antiallergy, reducing food hypersensitivities	(+) PPAR*γ* (-) IL-4, Th2, GATA3, p-STAT6,NF-AT	[268]
				5–30 *μ*M, treat, 48 h	PMA/ionomyc- in induced EL4 T cells			
		Hesperetin	Metabolic syndrome	10 *μ*M, treat, 72 h 50 *μ*M, treat, 24 h	3T3-L1 adipocytes	Decreased insulin, increased lipid accumulation, accelerated adipocyte differentiation, improved insulin resistance, lipid lowering	(+) PPAR*γ*, adiponectin, C/EBP*α*, glucose uptake (-) TG	[90, 91]
				1, 10, and 25 *μ*M, treat, 8 days	Adipocytes derived from hMSCs	Antiadipogenic effect, antiobesity	(+) atgl (-) PPAR*γ*, TG, C/EBP*β*, SREBP1C, perilipin, lipogenesis, fasn	[81]
			Liver diseases	50, 100,200 mg/kg, gavage, 7 days	CCl4-inducedALI C57BL/6J mice	Hepatoprotective effects	(+) PPAR*γ* (-) ALT, AST	[92]
				25 and 50 mg/kg, orally, 11 days	CYP-induced hepatotoxicity in rats	Anti-inflammatory, antioxidant, decreased lipid peroxidation	(+) PPAR*γ*, albumin, GSH, CAT, SOD, GPx (-) ALT, AST, *γ*GT, bilirubin, TNF-*α*, IL-1*β*, IL-6, MDA, iNOS, NO, NF-*κ*B	[97]
			Cancer	1-16 *μ*M, treat, 48 h	LPS-induced inflammation in RAW264.7 Cells	Anti-inflammatory	(+) PPAR*γ* (-) TNF-*α*, IL-1*β*, IL-6, JAK1, STAT1	[92]
				10–50 *μ*M, treat, 24 and 48 h	NALM-6 cells	Antiproliferative effects, increased subG1 phase cells, induced apoptosis	(+) PPAR*γ*, Bax, caspase 3, p53 (-) Bcl2, I*κ*B, NF-*κ*B	[96]
			Cardiovascular diseases	200 mg/kg, orally, 28 days	ISO-induced cardiac hypertrophy rat	Anti-inflammatory, antiapoptotic, antioxidant, attenuated pathological changes, improved cardiac hemodynamics	(+) PPAR*γ*, CAT, SOD, GSH, +dP/dt, -dP/dt, Bcl-2 (-) Heart weight, MDA, LDH, CK-MB, LVEDP, TNF-*α*, IL-6, NF-*κ*B, JNK, p-JNK, caspase-3	[93]
				100 mg/kg/day, orally,14 days	IR in diabetic rat	Cardioprotective, antiapoptotic, increased blood flow, antioxidant, anti-inflammatory, reduced edema, improved cardiac function	(+) PPAR*γ*, Bcl2, SOD, CAT, GSH, MAP, +dP/dt, -dP/dt, CK-MB, LDH, inotropic and lusitropic function (-) IS, Bax, TNF-*α*, MDA, LVEDP, thiobarbituric acid reactive	[94]
				50 and 100 mg/kg	AMI-rat	Antioxidation, anti-inflammatory, antiapoptotic, cardioprotective	(+) PPAR*γ*, Bcl2 (-) MDA, IS, HW/BW, CK-MB, TNF-*α*, IL-1*β*, IL-6, MCP-1, ICAM-1, caspase-3/9, p53, Bax	[95]
			Brain and nervous system diseases	100 mg/kg/day, orally, 8 weeks	Fluoride-induced neurobehavioral rat	Antioxidant, neuroprotective, increased fall time, improved neurobehavioral impairment	(+) PPAR*γ* receptor, preference index, GSH (-) Fluoride, MDA, DCF, AChE	[98]
		Apigenin	Dyslipidemia	50-200 *μ*M, treat, 24 and 48 h	3T3-L1 adipocytes	Inhibited early stage of differentiation, antiadipogenic, anti-inflammatory, antioxidant, reduced lipid accumulation, antiobesity	(+) G0/G1, S population (-) PPAR*γ*, C/EBP-*α*, SREBP-1c, FAS, TNF-*α*, IL-6, cell cycle progression, ROS	[99]
			Metabolic syndrome	15 and 30 mg/kg/day, SQI, 13 days	HFD-induced obese mice	Inhibited adipogenesis, anti-visceral obesity, reduced body weight	(+) STAT3 (-) PPAR*γ*, VAT, SAT, EAT, CD36	[100]
				10, 30, and 50 mg/kg, IP, 21days	HFD and ob/ob mice, RAW264.7 cells	Reduced liver and muscular steatosis in macrophage, improved glucose resistance, anti-inflammatory	(+) PPAR*γ*, MGL1/2, Ym1, Arg1, MMP-9, CD206 (-) ALT, AST, TC, TG, IL-12, TNF-*α*, IL-6, IL-1*β*,CCL2, CD80, MHCII, CCL3, CCL4, CCR2, p65	[101]
				30 mg/kg/day, IP, 3 weeks	HFD-induced mice NAFLD	Inhibited lipid accumulation, antioxidant, anti-inflammatory, attenuated liver steatosis	(+) Nrf2,Keap1, SOD, CAT, GSH-Px, GST, NQO1, GCLc, GCLm, GSTA2, GSTA4 (-) PPAR*γ*, PPAR*α*, TNF-*α*, MDA, MCP-1, F4/80, Cidea, Plin2, Fitm1, Fitm2, G0s2, Fabp1, Lpl, mCPT-1, PDK4, ACOX1, ACAA2, Fasn, SCD1, HMGCR, ACACA, Nrob2	[103]
			Liver diseases	20 and 40 mg/kg, gavage, three times a week, 8 weeks 20 and 40 mg/kg/day, gavage, 14 days	CCl_4_-induced mouse liver fibrosis BDL-induced mouse liver fibrosis	Alleviated liver fibrosis, suppressed autophagy, inhibited hepatic stellate cell activation, reduced cell viability, anti-inflammatory, deceased mean of integrated optical density of fibrotic and autophagy proteins, liver-protective	(+) PPAR*α*, MMP2, p62 (-) ALT, AST, ECM generation, HSCs, TIMP1, IL-1*β*, Col-1,*α*-SMA, Beclin-1, LC3, TGF-*β*1, p-p38	[102]
			Cardiovascular diseases	50-100 mg/kg/day, gavage, 4weeks	Renovascular hypertensive Rat	Cardioprotective, improved cardiac hypertrophy, regulated abnormal myocardial glucolipid metabolism	(+) PPAR*α*, CPT-1, PDK-4 (-) PPAR*γ*, SBP, angiotensin II, FFA, HIF-1*α*, GPAT, GLUT-4, Heart weight, Heart weight index	[104]
				75 mg/kg/day, orally, 14 days	MI-in diabetic rats	Attenuated myonecrosis, prevented edema, antiapoptotic, antioxidant, improved cardiac function, reinstated a balanced redox status, prevented hemodynamic perturbations	(+) PPAR*γ*, DAP, MAP, CAT, SOD, GSH, −LVdp/dt_min_, +LVdp/dt_min_ (-) Blood glucose, ST, SAP,HR, LVDEP, CK-MB, LDH, MDA	[105]
			Brain and nervous system diseases	20 mg/kg, intragastrically, 3 weeks	CUMS rat	Ameliorated behavioral abnormalities, decreased locomotor activity, inhibited microglia, antioxidant, anti-inflammatory, antidepressant	(+) PPAR*γ*, GSH, sucrose consumption, number of crossing (-) NLRP3, IL-1*β*, MDA, IL-18, CD11b, ASC, caspase-1	[106]
			Immunity	150–300 mg/kg, gavage, 28 days	Bleomycin-induced mouse pulmonary fibrosis	Antifibrotic, antioxidant	(+) PPAR*γ*, GSH, SOD, Smad-7, E-cadherin (-) NF-*κ*B, TGF-*β*1, MMP-9, vimentin	[107]
		Naringenin	Metabolic syndrome	3%, 11 weeks	Ovariectomized female mice	Inhibited lipid accumulation, reduced intra-abdominal and subcutaneous adiposity	(+) SREBF1, PPARGC1A, CPT1*α*, PGC1*α*, Pck2 (-) PPAR*α*^∗^, PPAR*γ*^∗^, MCP1/Ccl2, IL-6/Il6, leptin, glucose, insulin, TAG, LIPIDS,cholesterol,ACOX1	[108]
				12.5, 25, and 50 *μ*g/ml, treat, 24 h	HepG2 and HUVECs	Anti-inflammatory, reinforced metabolism, antihypercholesterolemia	(+) PPAR*γ*, LDLR, CYP7A1, SREBP2, I-*κ*B*α* (-) EL, CRP, TNF-*α*, ICAM-1, VCAM-1,ERK1/2, NF-*κ*B, p65	[109]
				100 mg/kg, orally, 4 weeks	Obese diabetic mice	Attenuated hypoglycemic, reduced obesity-related adipokine, antidiabetes	(+) PPAR*γ*, TIMP-1, CRP (-) Glucose	[115]
				100 and 200 mg/kg, IV, 16 weeks	STZ-induced diabetes mellitus rat	Increased body weight, enhanced blood glucose levels, ameliorated cognitive deficits, antioxidant, anti-inflammatory	(+) PPAR*γ*, GSH‑Px, GSH, SOD (-) Caspase-3, Caspase-9, MDA, TNF-*α*, IL-6	[116]
			Liver diseases	25, 50,100 mg/kg/d, orally, 28 days	HFD-STZ-induced type 2 diabetic rat	Antioxidant, anti-inflammatory, hepatoprotective, deceased kidney damage, antidiabetes, attenuated ER distension, preserved granule content, attenuated glomerular sclerosis, ameliorated hepatic steatosis	(+) PPAR*γ*, adiponectin, *β*-cell, HDL-C, P-IRS1(Tyr162), HSP-72, HSP-27, SOD, GSH-Px (-) Insulin resistance, TNF-*α*, IL-6, hyperinsulinaemia, CRP, NF-*κ*B, TC, TAG, LDL-C, NEFA, SREBP-1c, LXR*α*, dyslipidaemia, hyperglycaemia, TBARS	[117]
				0.003, 0.006, and 0.012% of diet, oral, 6 weeks	Rat	Hypolipidemic, antiadiposity, lowered adiposity, upregulated fatty acid oxidation	(+) PPAR*α*, CPT-1, UCP2 (-) TC, TG, free cholesterol	[110]
				30 mg/kg, oral gavage,14 days	HBx-induced hepatic lipid accumulation mice	Decreased hepatic lipid accumulation, inhibited hepatic adipogenic and lipogenic, prevented HBx-infected hepatic steatosis Increased fatty acid oxidation, deceased cholesterol and bile acid production, normalized lipid, inhibited HCV, decreased time-resolved fluorescence resonance energy transfer (TR-FRET)	(+) - (-) PPAR*γ*, LXR*α*, adipogenic, lipogenic, ALT,AST, TG, SREBP1c	[118]
				126 and 400 *μ*M, treat, 24	Huh7-rat hepatocytes cells		(+) PPAR*α*, PPAR*γ*, CYP4A, ACOX, UCP1, ApoAI, PGC1*α*, SREBP2 (-) LXR*α*,GAL4-fusion reporters, ABCA1, ABCG1, HMGR, FASN,LXRE, TG, bile acids, SREBP1, ApoB	[111]
			Renal diseases	25 or 75 mg/kg/d, 4 weeks	Diabetic nephropathy mice	Ameliorated the glomeruli and renal tubular injury, improved effect on diabetic nephropathy, alleviated the morphological changes, reduced the proliferation of NRK-52E cells	(+) PPAR*α*, PPAR*β*, PPAR*γ*, CYP4A-20-HETE (-) BUN, Scr, urinary albumin FBG	[119]
				0.01, 0.1, and 1 *μ*mol/L,	High glucose-induced proliferation and hypertrophy NRK-52E cells			
			Cardiovascular diseases	0.1, 1, and 10 *μ*mol/L, treat, 48 h	High glucose-induced cardiomyocyte hypertrophy H9c2 cells	Improved myocardial hypertrophy, antihypertrophic, cardioprotection	(+) PPAR*α*, PPAR*β*, PPAR*γ*, CYP2J3, 14,15-EET (-) -	[112]
			Brain and nervous system diseases	100 mg/kg/d, IP, 7 days	STZ-induced diabetic rat	Antioxidant, anti-inflammatory, antihyperglycemia, improved learning and memory performances, neuroprotective, reduced diabetes-associated cognitive decline	(+) PPAR*γ*, SOD (-) MDA, TNF-*α*, IL-1*β*, IL-6	[114]
				20, 40, and 80 mg/kg, orally, 28 days	Quinolinic acid-induced neurotoxicity rat	Neuroprotective effect, antioxidant, anti-inflammatory, decreased body weight and relative brain weight, antiapoptotic, decreased oxido-nitrosative stress, increased mitochondrial complex	(+) PPAR*γ*, SOD, GSH, NADH, complex I, complex II, complex III, complex-IV, Bcl-2 (-) Locomotor activity, rearing, grooming, neurological score, footprint analysis, grip strength, number of slips, TNF-*α*, IL-1*β*, IL-6, NF-*κ*B, MDA, NO, Bax , caspase 3	[113]
			Immunity	25, 50, and 100 mg/kg/d, orally, 7 days 20 *μ*M, treat, 1 h	DSS-induced ulcerative colitis in mice RAW264.7 cells	Anti-inflammatory, alleviated colitis outcomes, anti-UC activity, regulated ZO-1	(+) PPAR*γ* (-) Histological score, TNF-*α*, IL-1*β*, IL-6, NF-*κ*B p65, I*κ*B, p38, ERK, JNK, NLRP3, ASC, MAPK, caspase-1, DAI score, colonic shortening	[269]
		Catechins (catechin, EGCG, ECG, EGC, proanthocyanidins)	Metabolic diseases	50 and 100 mg/kg/d, gavage, 20 weeks	HFD-C57BL/6J mice	Decreased obesity and epididymal fat accumulation, increased free fatty acids excretion, increased *de novo* fatty acids synthesis genes, antihyperlipidemia, EGCG adipogenesis, lipogenesis, and lipolysis effects appear partially via AMPK activation in both subcutaneous and epididymal adipose tissues, antioxidant	(+) In subcutaneous adipose tissues: *PPARα*, *PPARγ*, *ACC1*, *FAS*, *SCD1*, *SREBP1*, *ACO2*, *MCAD*, *AP2, PGCLα*, lipolysis (*hsl, atgl*), lipid oxidization, In both: AMPK, HDL-C, FFA (-) In epididymal adipose tissue: *PPARγ*, *ACC1*, *FAS*, *SCD1*, *C/EBPB*, *SREBP1*, *hsl,* FASN, CPT1*α*, *PPARα*, *ACO2*, *MCAD*, *AP2, PGCLα*, UCP2 In both:TG, cholesterol, LDL-C, TAG	[120]
				100 *μ*M, treat, 2 days	DMI-induced 3T3-L1 preadipocytes	Inhibited cell proliferation, suppressed differentiation of 3T3-L1 preadipocytes, blocked adipocytes clonal expansion, lowering fat accumulation, antioxidant Promoted macrophage M2 polarization, suppressed M1 polarization, anti-inflammatory, ameliorated obesity-related inflammation, anti-inflammatory	(+) S-phase population (-) *PPARγ, C/EBPα, FoxO1*, PI3K/Akt, MEK/ERK, TAG, ROS, G_0_/G_1_ population	[121]
		Procyanidin B2		1-10 *μ*M, treat, 24 h	db/db C57BL/6J mice macrophages		(+) PPAR*γ*, CD36, ABCG1, CD206^+^, Arg1, Ym1, Fizz1 (-) CD86^+^, IL-6, TNF-*α*	[122]
					RAW264.7 cells			
				5, 10, 50, and 100 *μ*M, treat, 2 days	DMI-induced 3T3-L1 preadipocytes	Inhibited glucose uptake, reduced lipid accumulation, lowering adipokine secretion, blocked adipocyte's differentiation, suppressed maturation and functions of adipocyte, inhibited adipocytes secretory activity, antiobesity	(+) - (-) PPAR*γ*, FAS, P-FOXO1, PI3K/Akt, TNF-*α*, adiponectin, resistin, leptin	[123]
				25, 50, 75, and 100 *μ*M, treat, 24 h	Primary cultures of visceral preadipocytes from *Rattus norvegicus* strain Wistar	Inhibited preadipocytes differentiation into adipocytes, antiobesity, enhanced cell viability, inhibited preadipocytes differentiation	(+) Adiponectin (-) PPAR*γ*	[124]
				1, 5, and 10 *μ*M, treat, 14 days	Human adipose-derived stem cells	Increased cell proliferation, enhanced osteogenic differentiation, suppressed adipogenesis	(+) ALP, BSP, Runx2, OCN, STAT3 (-) PPAR*γ*, p-STAT3, C/EBP-*α*	[125]
				50, 100, 200, 300, 600, and 900 *μ*M, treat, 24 and 48 h	3T3-L1 preadipocytes	Inhibited preadipocytes differentiation, suppressed lipid decomposition, decomposed lipid, antidifferentiation, antiadipogenesis, antiobesity	(+) cAMP/PKA, HSL, ATGL (-) PPAR*γ*, C/EBP*α*, FAS, TG, C/EBP*β*, C/EBP*δ*, SREBP1C, PLIN	[126]
			Cancer	0-300 *μ*M, treat, 24 h	PANC1, TE-1, MCF-7, A2780	Induced cell death, anticancer, increased luciferase activity, PPAR*α* activation suppressed HO-1 induction by EGCG	(+) PPAR*α* (-) HO-1, Nrf2	[127]
			Cardiovascular diseases	1,10, and 50 *μ*M, treat, 0, 6, 12, and 24 h	HUVECs	Antioxidant, anti-inflammatory, protected vascular, increased luciferase activity	(+) PPAR*γ*, Pim-1, PPRE (-) -	[128]
			Brain and nervous system diseases	5–100 *μ*M, treat, 24 h	N2a-APP695 cells	Antiapoptotic, anti-inflammatory, antioxidant, neuroprotective	(+) PPAR*γ*, MnSOD (-) A*β*, BACE1, Bax, caspase-3, NF-*κ*B, ROS, MDA	[129]
			Renal diseases	25 and 50 mg/kg/d, gavage, 3 weeks	Crescentic GN 129/svJ mice	Anti-inflammatory, antioxidant, reduced mortality, ameliorated renal injury, improved renal histology and function	(+) PPAR*γ*, SIRT1, Nrf2, GSH, GCLC, GCLM, GPX1, NQO1 (-) p-Akt, p-JNK, p-ERK1/2, p-P38, proteinuria, serum creatinine, tubulointerstitial injury, glomerular in jury, lymphocyte, macrophages, MDA	[130]
Alkaloids	Berberine	Metabolic diseases	10, 20, and 40 mg/kg, orally,8 weeks	n-STZ-induced diabeticperipheral neuropathy rats	Neuroprotective, anti-inflammatory, antioxidant, inhibited aldose reductase, suppressed edema, ameliorated impaired allodynia, hyperalgesia, and nerve conduction velocity, decreasedoxido-nitrosative stress	(+) PPAR*γ*, IGF-1, BDNF, insulin, SOD, NO, GSH, Na-K-ATPase, Thr-172, AMPK (-) TG, cholesterol, TNF-*α*, IL-1*β*, IL-6, glycated Hb, glycosuria, MDA, PP2C-*α*, myelin degeneration, unmyelinated fibers	[132]
			75, 150, and 300 mg/kg, orally, 16-week	3T3-L1 diabetic adipocytes	Antihyperlipidemia, hypoglycemic, promoted differentiation, inhibited lipid accumulation, decreased white adipose tissue, increased adipocyte number	(+) PPAR*α*/*δ*/*γ*, CDK9, cyclin T1, aP2, P-TEFb, lipoprotein lipase (-) TNF-*α*, FFA	[133]
			300 mg/kg/d, gavage, 4 weeks	HFD rat	Lowering epididymal adipose tissue (EAT) and subcutaneous adipose tissue, alleviated liver steatosis, down-regulated lipogenesis, increased fatty acid oxidation, body weight lowering, promoted mitochondrial *β*-OX	(+) PPAR*α*, CPT-1*α*, Acox1, HDL, fatty acid *β*-OX, SIRT3, LCAD deacetylation (-) TG, TC, LDL, FBG, glucose, SREBP-1c, SCD1, FAS	[134]
			250 mg/kg/d, oral, 4 weeks 5 *μ*M, treat, 4 days	KKAy mice	Moderated glucose and lipid metabolism, ameliorated oral glucose tolerance and insulin sensitivity, increased energy dissipation	(+) PPAR*α*, GLUT4, MAPK14, MAPK8, UCP2, HNF4*α*, JNK, LDLR (-) PPAR*γ*, CEBP, PGC 1*α*, resistin, TG, TC, LDL-C, FBG	[135]
	13-Methylberberine			Mouse 3T3-L1 cells	Antiobesity, antiadipogenic, lipid-reducing	(+)AMPK, *Ddit3, Adrb2* (-) PPAR*γ*, C/EBP*α*, SREBP-1, *Adipoq*, *Fabp4*, *Pfkfb1,Slc2a4*, *Fasn, Cpt1a*, *Cpt2*, *Lipe*, *Mlycd*, ACC	[136]
			10 nM-10 *μ*M, treat, 6-72h; 60-300 mg/kg/d, intragastrically, 12 weeks	HepG2 cells, HFD rat	Lipid-lowering, improved lipid metabolism, hypolipidemic effect	(+) PPAR*α*, PPAR*δ*, CPT-I*α*, HDL (-) PPAR*γ*, TC, TG, LDL	[137]
			25, 50, amd 100 mg	NAFLD rat	Antihyperglycemic, improved insulin resistance, anti-inflammatory	(+) PPAR*γ*, HDL, insulin resistance (-) TC, TG, LDL, AST, ALT, TNF*α*, IL-6	[138]
		Cancer	50, 100, and 200 mg/kg, oral, 21 days	Bleomycin-induced pulmonary fibrosis in female ICR mice	Direct antifibrosis effect in a gut-dependent manner, anti-inflammatory, reduced edema, infiltration, parenchymal distortion, collapsed alveolar spaces, thicker alveolar membrane, and collagen deposition	(+) PPAR*γ*, HGF, PTEN, CD36, aP2 (-) -	[270]
		Renal diseases	1, 5, 10, 50, and 100 *μ*M, Treat, 24h	PA-induced lipotoxicity in HK-2 cells	Decreased lipid accumulation, anti-inflammatory, antiapoptosis, protected renal function, inhibited lipotoxicity	(+) PPAR*α*, CPT1, PERK (-) ER, FAS, ACC, LPL, CHOP, GRP78, TNF-*α*, IL-6, caspase3	[139]
		Cardiovascular	10, 30, and 100 *μ*mol/L, treat, 24 h	Angiotensin IV-induced VSMCs proliferation	Antiproliferative, inhibited OD value at the A490, decreased protein synthesis, regulated PPAR*α*-NOS-NO signaling pathway	(+) PPAR*α*, eNOS, NO (-) -	[140]
			15 and 30 mg/kg/day, intragastrically, 6 weeks	HSFD/streptozotocin rat	Protected diabetic cardiomyopathy, promoted glucose transport, alleviated cardiac lipid accumulation, increased cardiac output, decreased ventricular wall thickness, interventricular septum thickness, and collagen content	(+) PPAR*γ*, GLUT4, +d*p*/d*t*max, LVDP, fatty acid transport protein-1, fatty acid transport protein, fatty acid *β*-oxidase (-) PPAR*α*, −d*p*/d*t*max, TG, LVEDP, nonesterified FFA, fructosamine,fast blood glucose, glycated hemoglobin, glycosylated serum protein	[271]
			1 g/kg/day, gavage, 8 weeks 10, 50, and 100 *μ*M, treat, 1h 0.1-100 *μ*M, treat, 30 min	Collar placement-induced atherosclerosis in Apoe^−/−^ mice HUVECs	Antioxidant, increases carotid atherosclerotic plaque stability, decreased Oil Red^+^ lipid area, increased Sirius Red^+^ collagen area, protected endothelial function, attenuated endothelial dysfunction	(+) PPAR*γ*, collagen, NO, SOD (-) CD68^+^, vulnerability index, MDA, ROS	[272]
			1, 3, and 10 *μ*mol/L, treat, 0.5 h	HGI-induced cardiomyocyte hypertrophy in rat myocytes	Antihypertrophic, exhibited crosstalk between PPAR*α* /eNOS-NO transduction	(+) PPAR*α*, eNOS, NOS, NO (-) Cell surface area, protein level, ANF	[141]
		Brain and nervous system diseases		LPS-induced U251 cell death	Neuroprotective, increased cell viability	(+) PPAR*α*,CYP2J2, RXR*α* (-) -	[142]
Terpenoids	Cinnamic acid	Metabolic diseases	12.5, 25, 50, 100, and 200 *μ*M, treat, 24 h 20 mg/kg/BW, oral, 4 weeks	HepG2 cells *db/db* mice	Reduced lipid accumulation, suppressed hepatic lipogenesis, inhibited fatty acid intake, increased fatty acid oxidation, reduced body weight, liver mass and liver index, antidiabetic	(+) PPAR*α*, CPT1A, PGC1*α*, HDL, IHTG, ChREBP, BDK/PPM1K (-) PPAR*γ*, LXR, ACLY, ACC, FAS, SCD1, CD36, TG, glucose, SREPB1c	[144]
	Trans-Cinnamic acid	Brain and nervous system diseases	100 mg/kg/day, oral, 30 days	B6SJL-Tg male and female mice (5×FAD model of AD)	Stimulated lysosomal proteolysis, reduced A*β* burden in hippocampus and cortex, improved memory and behavioral deficit	(+) PPAR*α*, TFEB, cathepsin B, TPP1 (-) A*β* plaques, *β*CTF	[143]
			100 mg/kg/day, oral, 7 days	MPTP-induced PD in mouse	Neuroprotective, restored locomotor deficit, restored striatal neurotransmitters, protected dopaminergic neurons	(+) PPAR*α*, PPRE-luciferase, TH, TH+neuron, dopamine (-) -	[145]
	Glycyrrhizic acid (18*α*-GA + 18*β*-GA)	Metabolic syndrome	Different proportion, oral, 4 weeks	Ethanol-induced ALD in rat	Reduced ethanol-induced liver injury, decreased liver index, antioxidant, decreased hepatic steatosis, regulated lipid metabolism	(+) PPAR*α*, CPT1A, HDL, SOD, GSH (-) ALT, AST, ALP, GGT, TC, TG, LDL, MDA, SREBP-1c, ACC	[147]
			100 mg/kg, oral, 24 h	Rat	Improved serum lipid, increased insulin sensitivity, decreased blood glucose, regulated glucose homeostasis	(+) PPAR*γ,* LPL, HDL (-) HOMA-IR, TAG, TC, LDL, insulin	[148]
		Brain and nervous system diseases	5 mg/kg/day, injections, 6 days	Subarachnoid hemorrhage (SAH) rat	Antivasospastic, anti-inflammatory, increased body weight, decreased systolic blood pressure, regulated neuroinflammation	(+) PPAR*γ*, PPAR*δ* (-) IL-6, IL-1*β*, TNF-*α*, IL-8, CD45^+^, GOT, GPT, BUN/creatinine	[149]
	Oleanolic acid	Metabolic syndrome	1-50 *μ*M, treat, 48 h, 15 min (gene), 2 h (protein)	C2C12 muscle cells 3T3-L1 Murine fibroblasts	Antihyperglycemic, inhibited lipid accumulation, reduced cell functionality at 30 *μ*M and 50 *μ*M (toxic), improved insulin sensitivity	(+) PPAR*γ*, PPAR*α*, GLUT-4, GLUT-4 translocation, FATP-1, AdipoQ, ACSL (-) Lipid storage	[154]
			60 mg/kg, oral, 14 days	High fructose-fed rat	Antidiabetes, regulated glucose homeostasis	(+) PPAR*γ*-1, GlUT-4, glucose, glucose-1-phosphate, glucose-6-phosphate, ribose-5-phosphate (-) -	[152]
			0.1–50 *μ*M, treat, 24 h	High glucose-induced endothelial dysfunction in human vascular endothelial cells	Enhanced vasodilatation, increased arterial relaxation	(+) PPAR*δ*, NO, PDK4, ADRP, ANGPTL4, Akt-Ser^473^, eNOS-Ser^1177^ (-) -	[153]
		Liver disease	20, 40, and 80 mg/kg, injection, 3 days	Concanavalin A-induced acute liver injury in mice	Anti-inflammatory, attenuated autophagy and apoptosis, decreased necrotic area, congestion and lymphocytic accumulation, improved immunity	(+) PPAR*α*, Bcl-2 (-) JNK, TNF-*α*, IL-1*β*, IL-6, Bax, caspase-3, caspase-9, Beclin 1, LC3, ALT, AST, TRAF2	[155]
		Cardiovascular diseases	10 mg/kg (rabbit), 25 mg/kg (mice), oral, 5 weeks	Atherogenic diet-induced atherosclerosis in rabbit, C57BL/6J and LDLR^−/−^ mice	Reduced the thickness of intima, antiatherosclerotic, decreased lipid accumulation	(+) PPAR*γ*, AdipoR1, HDL-C (-) AdipoR2, TG, LDL-C, TC	[156]
	Ursolic acid	Metabolic syndrome	250 mg/kg/day, oral, 8 weeks	HFD rat	Ameliorated obesity and metabolic disorder, attenuated thermal hyperalgesia, decreased paw edema, anti-inflammatory, reduced body weight, inhibited spinal cord inflammation	(+) PPAR*α*, adiponectin, I*κ*B*α* (-) Insulin, cholesterol, leptin, IL-1*β*, TNF-*α*, COX-2, iNOS, NF-*κ*B p65	[160]
			25 mg/kg/d, oral, first 6 h	Tern-type diet-induced hyperglycemia in rabbit	Improved hypolipidemic and antiatherosclerosis efficacy, reduced lesions area, increased lumen area	(+) PPAR *α* (-) TG, cholesterol, VCAM-1	[158]
		Liver disease	5-100 *μ*M, treat	HepG2 cells	Regulated lipid metabolism, enhances PPAR*α* binding to PPRE	(+) PPAR*α*, PPRE, fatty acid uptake, FATP4, ACS, CPT1, ACOX (-) TG, cholesterol, SCD1, SREBP1c	[163]
		Brain and nervous system diseases	25 mg/kg/d, gavage, 120 days	EAE and cuprizone-induced demyelination Female C57BL/6J mice	Enhanced remyelination, anti-inflammatory, promoted myelin repair, immunomodulatory, repaired neural, anti-multiple sclerosis, reduced remyelinated axons G-ratio, neuroreparation	(+) PPAR*γ*, CREB, CNTF, MBP, CC1, GFAP (-) CD45^+^, A2B5, CD11b^+^, CD11c^+^, IFN-*γ*^+^, IL-17^+^, GM-CSF^+^, CD4^+^ T cell, Th17, Th1	[164]
			5, 10, and 20 mg/kg, gavage, 0.5, 24, and 47 h after reperfusion	Cerebral ischemia/reperfusion rat	Neuroprotective, improved neurological deficit score and general condition, decreased median neurological deficit score, alleviated histological damage, increased intact neuron number, attenuated cerebral ischemia/reperfusion injury	(+) PPAR*γ*, TIMP1 (-) MMP2, MMP9, MAPKs, infarct size, pERK1/2, pJNK1/2, pp38	[159]
		Respiratory diseases	2 and 20 mg/kg, orally, 3 times a week for 5 weeks	Allergic asthma mouse	Suppressed eosinophil infiltration, anti-inflammatory, antiasthma, decreased blood basophil and eosinophils, reduced airway inflammation, reduced total bronchoalveolar lavage fluid cells, decreased eosinophils in bronchoalveolar lavage fluid, acted as antagonist of Th2 and Th17	(+) PPAR*γ*, Foxp3 (-) IL-5, IL-13, IL-17, GATA-3, STAT6, NF-*κ*B, CCR3, ovalbumin-IgE, CD4^+^	[165]
			50 mg/kg/d, gavage, 4 weeks	PAH-induced RV in Sprague Dawley rat	Improved RV function, attenuates RV hypertrophy, inhibited RV fibrosis, reduced apoptosis, regulated metabolic abnormalities	(+) PPAR*α*, CPT1b (-) ANP, BNP, TGF-*β*1, COL3A1, COL1A1, collagen, Bax, apoptotic cell	[162]
		Immunity	250 mg/kg/day, orally, 8 weeks	HFD-induced inflammation	Anti-inflammatory, ameliorated obesity, regulated metabolic disorder, prevented thermal hyperalgesia and paw edema, restored spinal cord inflammatory response	(+) PPAR*α*, I*κ*B*α* (-) NF-*κ*B, BW, IL-1*β*, TNF-*α*, COX-2, iNOS	[160]
	6-Shogaol	Cancer	0.1–100 *μ*M, treat, 72 h	MCF-7 and HT29 cells	Inhibited breast and colon cancer cell proliferation, antitumor effects, induced apoptosis and cell cycle arrest, exhibited binding to PPAR*γ*	(+) PPAR*γ*, PPRE, Cdc2, Cdc25C, caspase3, caspase 9, CYP1A1, CDKN1A, GADD45A, Bax (-) NF-*κ*B, G2/M cell cycle, G1, ASCL1, CAV1, CXCL12, Bcl-2, Bcl-X_L_, p65, PPAR*γ*si-1, PPAR*γ*si-2	[273]
		Brain and nervous system diseases	5, 10, and 20 *μ*g/mL, 1h before LPS	LPS-activated BV2 microglia	Antitumor, anti-inflammatory, protected neurodegeneration	(+) PPAR*γ* (-) TNF-*α*, IL-1*β*, IL-6, PGE2, I*κ*B*α*	[167]
Fatty acids	Oleic acid	Metabolic syndrome	50, 100, and 200 *μ*M, treat, 24h	Aorta smooth muscle cells	Antioxidant, anti-inflammatory, protected coronary artery	(+) MMP-1, MMP-3, iNOS, NO, TGF-*β*1, NF-*κ*B (-) PPAR*γ*, SIRT1	[169]
		Liver disease	0.1-1 mM, treat, 24h	HepG2 steatotic cells	Regulated insulin sensitivity, induced lipid accumulation, increased *β*-oxidation, enhanced insulin sensitivity, antisteatosis	(+) PPAR*δ*,GPR40, Ca^2+^ influx, PLC (-) PTEN	[171]
		Renal diseases	1.25 *μ*M, treat, 45 min	OGD/R-HK-2 cells	Attenuated apoptosis, increased cell viability, restored nuclei shape, protected against ischemia/reperfusion	(+) PPAR*γ*, p-Akt, p-GSK3*β*, cytochrome C, AIF (-) Caspase-3, Bax	[274]
		Brain diseases	10 and 30 mg/kg, intraperitoneally, 90min after model	Middle cerebral artery occlusion-induced ischaemic stroke in rat	Neuroprotection, anti-inflammatory, anti-cerebral ischaemic, enhanced functional outcomes, reduced infarct volume, increased neuronal densities	(+) PPAR*γ*, neuronal densities (-) Infarct size, COX-2, TNF-*α*, iNOS	[275]
	n-3 polyunsaturated fatty acid	Liver disease	0.2 g/kg/d, injection, 5 days	Hemorrhagic shock/resuscitation mice	Improved lipid oxidation in the liver	(+) PPAR*α*, CPT-1A, FATP-1 (-) TG	[174]
		Cancer	50 and 120 *μ*M, treat, 24, 48, and 72 h	MGC and SGC cells	Anticancer, anti-inflammatory, anticachectic, inhibited gastric tumor cells	(+) PPAR*γ*, C/EBP*α* (-) TNF-*α*, VEGF	[175]
	n-6	Cardiovascular diseases	50 mg/kg, orally,4 weeks	EC_80_-induced thrombin in mice	Inhibited arterial thrombosis, antiplatelet	(+) PPAR*α* (-) Platelet aggregation, calcium mobilization, PKC, dense granule secretion, collagen	[176]
	n-3	Immunity	20 mg/kg/day, intragastric, 60 days	TNBS-induced Crohn's disease in rat	Anti-inflammatory, immunity, attenuated colonic inflammation	(+) PPAR*γ* (-) NFAT, TC, IL-6, IL-12, IL-2, IL-4, TNF-*α*	[177]
			5 kg FO, oral, 14 days	Mastitis rat	Decreased mammary inflammation	(+) PPAR*γ*, IL-10 (-) PMN, XOR, IL-1*β*, TNF-*α*	[178]

(+): Increasing or activation of target; (-): Decreasing or inhibition of target; ∗: not significant (or no effect).

**Table 2:** Summary of clinical studies of phytochemicals on the PPAR family in diseases.

Phytochemicals	Disease	Dose/route of administration	Assay	Protective effect	Mechanism	Ref.
Nano-curcumin	Diabetes on hemodialysis (HD)	80 mg/day, capsule, 12 weeks (RCT)	Gene expressions in PBMCs, blood sample	Antioxidant, antidiabetic, anti-inflammatory	(+) PPAR*γ* mRNA, LDLR mRNA, HDL-cholesterol, TAC, total nitrite level (-) FPG, insulin level, TC, TG, VLDL-cholesterol, LDL-cholesterol, total-/HDL-cholesterol ratio, hs-CRP, MDA	[201]
Curcumin	Polycystic ovary syndrome	500 mg/day, supplementary, 12 weeks (RCT)	Fasting blood sample, insulin and lipid metabolism gene expressions	Antiobesity, antidiabetic, lipid lowering	(+) PPAR*γ* mRNA, LDLR mRNA, HDL cholesterol (-) FPG, insulin level, HOMA-IR, TC, LDL-cholesterol, total-/HDL-cholesterol ratio	[202]
Resveratrol + curcumin	Postprandial inflammation response in high-fat meal	100/50 mg (Res/Cur), 2 capsule, 30 min before consuming the high-fat meal (RCT)	Blood sample, inflammatory markers, adhesion molecules, NF*κ*B1, and PPAR*α*	No impact on the postprandial inflammation response, have only small effects on endothelial function	(+) – (-) sVCAM-1 iAUC ∗: *PPARα*and *NFκB1* not changed	[203]
Resveratrol	Type 2 diabetes mellitus and coronary heart disease	500 mg/day, capsule, 4 weeks (RCT)	Fasting blood sample, lipid, inflammation and oxidative markers, related gene expression	Antidiabetic, antioxidant, regulated dyslipidemia ∗Not effect on inflammatory markers	(+) PPAR*γ*, SIRT1, QUICKI, HDL-C, TAC (-) FPG,insulin, HOMA-IR, TC/HDL, MDA	[276]
Naringenin	Diabetes	150 mg, capsule, 3times/day, 8 weeks (a case)	Blood sample, respiratory quotient, insulin and metabolic markers	Reduced body weight and insulin resistance, increased metabolic rate	(+) PPAR*α*,PPAR*γ*, serum glucose,UCP1, CPT1*β* (-) HOMA-IR, LDL-C	[204]
Epigallocatechin gallate	Obesity	150 mg, capsule, twice/day, 8 weeks (RCT)	Blood sample (enzyme and hormone assay), gene expression in adipocytes	Decreased blood pressure, no effects on obesity, lipolysis and browning of human white adipocytes	(+) - (-) TG,serum kisspeptin ∗ not effect on PPAR*γ* and UCP1 expressions	[205]

(+): Increasing or activation of target. (-): Decreasing or inhibition of target.

## Data Availability

There is no raw data associated with this article.

## Abbreviations

A*β*: *β*-Amyloid peptides
ABCA1: ATP-binding cassette transporter A1
ABCG1/5/8: ATP-binding cassette transporters G1/5/8
ACAA2: Acetyl-coenzyme A acyltransferase 2
ACACA: Acetyl-CoA carboxylase alpha
ACADL: Acyl-CoA dehydrogenase long chain
ACAT: Acyl-CoA cholesterol acyl transferase
ACC: Acetyl-CoA carboxylase
AChE: Acetyl cholinesterase
ACOT: Acyl-CoA thioesterase
ACOX1: Acyl-CoA Oxidase 1
ACSL: Long-chain acyl-CoA synthetase
AdGFP: AdCMV-GFP control vector
Agpat2: 1-Acylglycerol-3-Phosphate O-Acyltransferase 2
AIF: Apoptosis-inducing factor
Akt: Protein kinase B
ALB: Albumin
ALOX5: Arachidonate 5-lipoxygenase
ALP: Alkaline phosphatase
ALT: Alanine transaminase
AMPK: AMP-activated protein kinase
ANF: Atrial natriuretic factor
Ang II: Angiotensin II
ANP: Atrial natriuretic peptide
AP-1: Activator protein 1
APG-7: Autophagy protein 7
APOC3: Apolipoprotein C3
Arg1: Arginase
AOPP: Advanced oxidation protein product
AOX: Acyl-CoA oxidase
Apo-AI: Apolipoprotein A-I
Apo-AII: Apolipoprotein A-II
apoE^−/−^: Apolipoprotein E-defcient
*β*3AR: *β*3-Adrenergic receptor
ASC: Apoptosis-associated speck-like protein
Atgl: Adipose triglyceride lipase
ATGL: Adipose triglyceride lipase
ATP: Adenosine triphosphate
AUC: Area under curse
BACE1: *β*-Site amyloid precursor protein-cleaving
BAT: Brown adipose tissue
BDL: Bile duct ligature
BDNF: Brain-derived neurotrophic factor
BMI: Body mass index
BNP: Brain natriuretic peptide
BSP: Bone sialoprotein
BUN: Blood urea nitrogen
cAMP: Cyclic adenosine monophosphate
CaMKII: Ca^+2^/Calmodulin-dependent protein kinase II
CAT: Catalase
CatB/L: Cathepsin B/L
CD36: Scavenger receptor (class B)
CD11c: Scavenger receptor
CD206: Cluster of differentiation 206
cdk1: Cyclin-dependent kinase 1
C/EBP*α*: CCAAT-enhancer-binding protein *α*
CE: Esterified cholesterol
Cel: Carboxyl ester lipase
CETP: Plasma cholesterol ester transferase
Cfd: Complement factor D
ChAT: Cholineacetyltransferase
ChE: Cholesterol efflux
CHOP: C/EBP-homologous protein
Cidea: Cyclic adenosine monophosphate
CK-MB: Creatine kinase on myocardial bundle
CNTF: Ciliary neurotrophic factor
Col1*α*1/2: Collagen type I alpha-1/2
COX1: Cytochrome C oxidase subunit 1
COX-2: Cyclooxygenase-2
CPT1*β*: Carnitine palmitoyl-transferase 1 *β*
CREB: cAMP response element-binding
CRP: C-reactive protein
CTGF: Connective tissue growth factor
CVD: Cardiovascular disease
CVF: Collagen volume fraction
cTnT: Cardiac troponin T
Cyt C: Cytochrome CDAB
CUMS: Chronic unpredictable mild stress
DAP: Diastolic arterial pressure
DCF: 2′,7′-Dichlorofluorescein
DGAT1/2: Diacylglycerol O-Acyltransferase 1/2
DsbA-L: Disulfide-bond A oxidoreductase-like protein
EAT: Epididymal adipose tissues
ECM: Extracellular matrix
eGFR: Estimated glomerular filtration rate
Ehhadh: Enoyl-CoA hydratase and 3-Hydroxyacyl CoA dehydrogenase
EMT: Epithelial-to-mesenchymal transition
ER: Endoplasmic reticulum
ERK: Extracellular signal-regulated kinase
eIF2*α*: Phosphorylation of eukaryotic initiation factor-2*α*
eNOS: Endothelial nitric oxide synthase
ERR-1*α*: PPAR*α*–estrogen-related receptor
FABP1/4: Fatty acid binding protein 1/4
FAS: Fatty acid synthase
FBG: Fasting blood glucose
FC: Free cholesterol
FEUA: Fractional excretion of uric acid
FFA: Free fatty acid
FINS: Fasting insulin
FN: Fibronectin
Fitm1/2: Fat-induced transcript 1/2
Fizz1: Found in inflammatory zone
FoxO1: Forkhead transcription factor O 1
FPG: Fasting plasma glucose
FSI: Fasting serum insulin
FSP27: Fat-specific protein 27
FS: Fractional shortening
GA: Glycyrrhizic acid
GCLC: Glutamatecysteine ligase catalytic subunit
GCLm: Glutamyl cysteine ligase Modifier Subunit
GIR60–120: Glucose infusion rate between the 60th and 120th minute
GFAP: Glial fibrillary acidic protein
GK: Glycerol Kinase
GLUT-4: Glucose transporter-4
GOT: Glutamic-oxaloacetic transaminase
GPAT: Glycerol-3-phosphate acyltransferase
G3PDH: Glyceraldehyde-3-phosphate dehydrogenase
GPR120: G protein-coupled receptor 120
G6Pase: Glucose 6-phosphatase
GPT: Glutamate pyruvate transaminase
GPX3: Plasmatic glutathione peroxidase
GRP78: 78 kDa glucose-regulated protein
GSH: Glutathione
G0S2: G0/G1 switch gene 2
GST: Glutathione S-transferase
*γ*GT: Gamma glutamyl transferase
HbA1c: Hemoglobin A1c
HDL-C: High-density lipoprotein cholesterol
HFHS-D: High-fat high-sucrose diet
HGF: Hepatocyte growth factor
HIF-1*α*: Hypoxia inducible factor-l*α*
HK-2: Normal human kidney epithelial
HMGCR: 3-Hydroxy-3-methylglutaryl-CoA reductase
4-HNE: 4-Hydroxynonenal
HO-1: Heme oxygenase-1
H2O2: Hydrogen peroxide
HOMA-IR: Homeostatic model assessment-insulin resistance
HR: Heart rate
HSCs: Hepatic stellate cells
hs-CRP: High-sensitivity C-reactive protein
HSL: Hormone-sensitive lipase
Hsp70: Heat shock protein70
HUVECs: Human umbilical vein endothelial cells
iAUC: Incremental AUC
ICAM-1: Intracellular adhesion molecule-1
IFN-*γ*: Interferon gamma
IKK: I*κ*B kinase
IL-1: Interleukin-1
IL-6: Interleukin-6
IL-10: Interleukin-10
IL-13: Interleukin-13
iNOS: Inducible nitric oxide synthase
iROS: Intercellular reactive oxygen species
IS: Infarct size
IVSd: End-diastolic interventricular septal thickness
JNK: c-JUN N-terminal kinase
Keap1: Kelch-like ECH-associated protein 1
LAMP-1/2: Lysosome-associated membrane protein 1/2
LC3: Protein light chain 3
LDH: Lactate dehydrogenase
LDL-C: Low-density lipoprotein cholesterin
LDLR^−/−^: Lack the LDL receptor
LPAAT*θ*: Lysophosphatidic acid acyltransferase-*θ*
LPL: Lipoprotein lipase
LPIN1: Lipin1
L-PK: L-Pyruvate kinase
LSR: Lipolysis-stimulated receptor
LXR: Liver X receptor
+LVdp/dt_min_: Maximal positive rate of left ventricular pressure
−LVdp/dt_min_: Maximal negative rate of left ventricular pressure
LVIDd: Left ventricular end-diastolic internal diameter
LVEDP: Left ventricular end diastolic pressure
LVPWd: Left ventricular end-diastolic posterior wall thickness
MAP: Mean arterial pressure
MAPK: Mitogen-activated protein kinase
MALAT1: Metastasis-associated lung adenocarcinoma transcript 1
MBP: Myelin basic protein
MCAD: Mitochondrial medium-chain acyl-CoA dehydrogenase
MCP-1: Monocyte chemoattractant protein-1
MDA: Malondialdehyde
ME: Malic enzyme
MI: Myocardial infarction
MMPs: Matrix metalloproteinases
mTOR: Mammalian target of rapamycin
MUFA: Monounsaturated fatty acids
NEFAs: Nonesterifed fatty acids
NF-*κ*B: Nuclear factor-*κ*B
NLRP3: NOD-like receptor family pyrin domain containing 3
NO: Nitric oxide
NOX: NADPH oxidase
NPT: Non-protein thiol
NQO-1: NADPH quinone oxidoreductase
Nrf2: Nuclear factor erythroid 2-related factor 2
NRF-1: Nuclear respiratory factor-1
NRK-49F: Rat renal interstitial fibroblasts
O_1_: Immature OL
O_4_: Pre-OL
OAT1: Organic anion transporter 1
OCN: Osteocalcin
OCTN2: Organic cation transporter 2
OP: Oligodendrocyte progenitor
oxLDL: Oxidized low-density lipoprotein
PAI-1: Plasminogen activator inhibitor
PBEF: Pre-B cell colony enhancing factor
PCNA: Proliferating cell nuclear antigen
PcSK9: Proprotein convertase subtilisin/kexin type 9
PDE/cAMP: Phosphodiesterase/Cyclic adenosine monophosphate
PDGF-*β*: Platelet-derived growth factor subunit B
PDK-4: Pyruvate dehydrogenase kinase-4
PERK: Prospective evaluation of radial keratotomy
PGC-1*α*: Peroxisome proliferator-activated receptor gamma coactivator 1-alpha
PGE2: Prostaglandin E2
PI3K: Phosphatidylinositol-3 kinase
PKA: Protein kinase A
Plin2: Perilipin 2
Pnpla2: Adipose triglyceride lipase
PON-1/2/3: Paraoxonase-1/2/3
PPAR*α*: Peroxisome proliferator-activated receptor alpha
PPAR*γ*: Peroxisome proliferator-activated receptor gamma
PPAR*δ*: Peroxisome proliferator-activated receptor delta
PP2C-*α*: Protein phosphatase 2C-*α*
PPRE: Peroxisome proliferator response elements
Prdm16: PR domain containing 16
PT: Protein thiol
P-TEFb: Positive transcription elongation factor b
PTEN: Phosphatase and tensin homolog
PUFA: Polyunsaturated fatty acids
QUICKI: Quantitative insulin sensitivity check index
RAGE: Advanced glycosylation end products receptor
rIR: Insulin receptor
ROS: Reactive oxygen species
RST: Renal-specific transporter
RUNX2: Runt-related transcription factor 2
RXR: Retinoid X receptor
SAH: Subarachnoid hemorrhage
SAP: Systolic arterial pressure
SAT: Subcutaneous adipose tissue
SBP: Systolic blood pressure
SCD-1: Stearoyl CoA desaturase-1
Scr: Serum creatinine
SFA: Saturated fatty acids
SIRT1: NAD-dependent protein deacetylase
S6K1: Ribosomal S6 kinase 1
*α*-SKA: *α*-Skeletal actin
SLU: Selective lipid uptake
*α*-SMA: Alpha smooth muscle actin
SOCS3: Suppressors of cytokine signaling 3
SOD: Superoxide anion dismutase
SR-A: Scavenger receptor-A
SR-BI: Scavenger receptor class B type I
SQI: Subcutaneous injection
SREBP-1: Sterol regulatory element-binding protein-1
STAT3: Signal transducer and activator of transcription 3
Surf: Surfeit locus protein
sVCAM-1: Soluble vascular cell adhesion molecule-1
TAG: Triacylglycerol
T-AOC: Total antioxidative capability
TBARS: Thiobarbituric acid reactive substances
3T3-L1 cells: Mouse preadipocytes
TC: Total cholesterol
TERT: Antitelomerase reverse transcriptase
Tfam: Transcription factor A
TG: Triglyceride
TGF: Transforming growth factor
TIMP-2: Tissue inhibitor of metalloproteinase
TH: Tyrosine hydroxylase
TNF-*α*: Tumor necrosis factor alpha
Tmem26: Transmembrane protein 26
TPP1: Tripeptidyl-peptidase 1
TRAF2: TNF receptor associated Factor 2
Treg: Regulatory T cells
TR-FRET: Time-resolved fluorescence resonance energy transfer
TXNIP: Thioredoxin interacting protein
UA: Uric acid
UCP 1/2: Uncoupling protein 1/2
VAT: Visceral adipose tissue
VLDL-cholesterol: Very low-density lipoprotein-cholesterol
VSMCs: Vascular smooth muscle cells
XDH: Xanthine dehydrogenase
XO: Xanthine oxidase
XOR: Xanthine oxidoreductase
ZO-1: Zonula occludens-1.
